# Response adaptive designs for Phase II trials with binary endpoint based on context-dependent information measures^☆^

**DOI:** 10.1016/j.csda.2021.107187

**Published:** 2021-06

**Authors:** Ksenia Kasianova, Mark Kelbert, Pavel Mozgunov

**Affiliations:** a National Research University Higher School of Economics, Moscow, Russia; b Department of Mathematics and Statistics, Lancaster University, Lancaster, UK

**Keywords:** Experimental design, Phase II clinical trial, Information gain, Small population trials, Weighted information

## Abstract

In many rare disease Phase II clinical trials, two objectives are of interest to an investigator: maximising the statistical power and maximising the number of patients responding to the treatment. These two objectives are competing, therefore, clinical trial designs offering a balance between them are needed. Recently, it was argued that response-adaptive designs such as families of multi-arm bandit (MAB) methods could provide the means for achieving this balance. Furthermore, response-adaptive designs based on a concept of context-dependent (weighted) information criteria were recently proposed with a focus on Shannon’s differential entropy. The information-theoretic designs based on the weighted Renyi, Tsallis and Fisher informations are also proposed. Due to built-in parameters of these novel designs, the balance between the statistical power and the number of patients that respond to the treatment can be tuned explicitly. The asymptotic properties of these measures are studied in order to construct intuitive criteria for arm selection. A comprehensive simulation study shows that using the exact criteria over asymptotic ones or using information measures with more parameters, namely Renyi and Tsallis entropies, brings no sufficient gain in terms of the power or proportion of patients allocated to superior treatments. The proposed designs based on information-theoretical criteria are compared to several alternative approaches. For example, via tuning of the built-in parameter, one can find designs with power comparable to the fixed equal randomisation’s but a greater number of patients responded in the trials.

## 1 Introduction

Consider a Phase II clinical trial with two independent treatment arms, $A_{1}$ and $A_{2}$, associated with unknown efficacy probabilities of a binary response. The goal is to find the superior treatment having the highest probability of the efficacy response. Assume that a rare disease trial is considered and an investigator would like to assign as many patients as possible to the superior arm motivated by the ethical reasons (Williamson et al., 2017). Assume that 10 patients were assigned to each arm and 4 and 6 responses were observed, respectively. Then, a typical question in a sequential trial is: “Which treatment arm should be assigned to the next patient?”. There are two main strategies to answer this question.

The first one is the “best intention strategy” which is to choose the arm with the highest probability of success. Assume that in the example above, the probabilities $P_{1}$ and $P_{2}$ are considered as random variables with Beta distributions B(4,6) and B(6,4), and one uses the mean as the point estimate: ${\overset{ˆ}{p}}_{1}=0.4$ and ${\overset{ˆ}{p}}_{2}=0.6$. Then, following this strategy, the next patient should be assigned to arm $A_{2}$ as it corresponds to the greater success estimate. While this approach is expected to result in a higher number of responses, it can also lead to a lower number of observations on other arms and an unacceptably poor performance in terms of the statistical power (Villar et al., 2015a).

The second strategy is to use measures of the statistical information and to assign the next patient to the arm about which an investigator knowns “less” (Cover and Thomas, 2012, Sebastiani and Wynn, 2000, Sebastiani and Wynn, 2001). For instance, applying the Shannon differential entropy

(1) $$h\left(f\right)=-\int_{0}^{1}f\left(p\right)\log f\left(p\right)dp$$

to Beta distributions $f_{1}\left(p_{1}\right)$, $f_{2}\left(p_{2}\right)$ as above, one can find that $h\left(f_{1}\right)=h\left(f_{2}\right)$. This equality means that both arms correspond to the same amount of uncertainty (Kelbert and Mozgunov, 2015b) and the next patient can be assigned to either of these arms. The same conclusion can be made with other measures of information, e.g., the Fisher information (Kelbert and Mozgunov, 2015a, Suhov et al., 2016). This approach is expected to lead to a high statistical power, but a low number of patients on the superior treatment as it does not account for the fact that one would like to maximise the number of treated patients. This shortcoming is a consequence of “standard” information measures being *context-free* meaning that they do not depend on the nature of outcomes p, but only on their probability density f(p).

Overall, it is desirable to balance these two strategies to achieve a high statistical power and a high average number of patients that respond to the treatment. This problem is known as an “exploration vs exploitation” (also known as “learn versus earn” trade-off Azriel et al., 2011). It was recently advocated in Williamson et al. (2017) that designs maximising the expected number of responses in small populations trials should get more attention. As a result, response-adaptive methods based on optimal multi-arm Bandit (MAB) approaches are starting to be considered more commonly as an option for Phase II clinical trials. Although MAB designs outperform other well-established methods of randomisation in terms of the expected number of responses (e.g. the fixed randomisation), they can suffer from a low statistical power for testing comparative hypotheses as described above. Thereby, some modifications have been proposed to achieve a better balance of the two objectives, see, e.g. Berry, 1978, Villar et al., 2015a, Villar et al., 2015b, Villar and Rosenberger, 2018 and Williamson et al. (2017).

As an alternative to these MAB approaches, a response-adaptive design based on a novel information-theoretical criterion for the arm selection in sequential experiments was proposed (Mozgunov and Jaki, 2020). The criterion is constructed using the so-called *context-dependent* measure of information accounting for both the uncertainty about the parameter of interest (e.g. probability of response) and the nature of outcomes. Thus, the criterion can carry the information that an investigator would like to maximise the number of patients that respond to the treatment.

Specifically, Mozgunov and Jaki, 2019, Mozgunov and Jaki, 2020 proposed to use the information-theoretical criterion based on the weighted Shannon differential entropy (WDE)

(2) $$h^{\phi }\left(f\right)=-\int_{0}^{1}\phi \left(p\right)f\left(p\right)\log\left(f\left(p\right)\right)dp$$

where $\phi :R\mapsto R^{+}$ is a positive weight function that answers the question “Which outcomes are more desirable?”. Then, the leading term of the information gain *asymptotic expansions*

(3) $$\delta_{S}=h^{\phi }\left(f\right)-h\left(f\right)$$

was proposed as the selection criterion to be used for the sequential allocation of patients. It was shown that the response-adaptive methods that use derived information-theoretic criterion to govern the treatment selection allow to achieve various balances “exploration vs. exploitation” trade-off via tuning of the weight function. It was found that the resulting designs for tuned values of the parameters can have better operating characteristics in comparison to other competitive approaches.

In this work, we extend the response-adaptive procedures based on a information-theoretical criteria and investigate whether other information measures, namely the Renyi, Tsallis and Fisher informations, can provide a better exploration versus exploitation trade-off compared to the previously studied Shannon’s entropy in the settings of clinical trials with a binary endpoint. Formally, the weighted Fisher Information (WFI)

(4) $$I^{\phi }\left(\theta \right)=E\left(\phi \left(P\right){\left(\frac{\partial }{\partial \theta }\log\left(f\left(P,\theta \right)\right)\right)}^{2}|\;\theta \;\right),$$

the weighted Renyi Information (WRI)

(5) $$H_{\nu }^{\phi }\left(f\right)=\frac{1}{1-\nu }\log\int_{0}^{1}\phi \left(p\right){\left(f\left(p\right)\right)}^{\nu }dp,\nu \geq 0,$$

and the weighted Tsallis Information (WTI)

(6) $$T_{q}^{\phi }\left(f\right)=\frac{1}{q-1}\left(1-\int_{0}^{1}\phi \left(p\right){\left(f\left(p\right)\right)}^{q}dp\right),q\in R,$$

are considered and response-adaptive designs are constructed based on the corresponding information gains. While the Fisher information is widely known in statistics, the other two are often used in physics. The use of additional parameters ν and q in the Renyi and Tsallis entropies, respectively, might widen the range of possible outcomes, which can improve the balance between gaining statistical power while preserving the number of patients getting superior treatment.

While Mozgunov and Jaki (2020) propose both asymptotic and exact maximum gain criteria, their analysis was primarily focused on the asymptotic one. In this paper, both the asymptotic and exact expressions for the information gain will be considered and and compared to each other for each entropy measure. Although the leading term of an asymptotic expression results in a simple and straightforward criterion, it is a “truncated” version of the information gain. Hence, it is investigated whether the use of asymptotic criteria instead of the exact one leads to any noticeable changes the designs’ properties.

Furthermore, the designs based on information-theoretical criteria are compared to several alternative approaches, namely the fixed equal randomisation (FR), optimal constrained randomised dynamic programming (CRDP) (Williamson et al., 2017) and optimal dynamic programming (DP), in a comprehensive simulation study in the setting of a small population Phase II clinical trial. It will be shown that tuning of the built-in parameters allows to find the designs comparable to FR performance in terms of power, but a greater number of patients assigned to the superior treatment, and the designs with comparable or superior to CRDP performance in terms of both of these characteristics.

The rest of the work proceeds as follows. The derivation of the novel criterion, together with the proposed selection criteria, is given in Section 2. The comparison of the asymptotic and exact criteria using different measures of information in a comprehensive study is presented in Section 3. The comparison to the recently proposed designs is given in Section 4. Section 5 concludes with the discussion.

## 2 Methods

### 2.1 Derivation of context-dependent information measures

Consider a random variable (r.v.) P which corresponds to the probability of response for an arm. P is assumed to have a prior Beta distribution, P∼B(υ+1,β−υ+1), υ>−1,β−υ>−1. Suppose that an arm was assigned to n patients and x responses were observed. Then, the posterior PDF of P takes the following form

(7) f(n)(p|x)=(n+β+1)n+βx+υpx+υ(1−p)n−x+β−υ.

Let us assume $\frac{x}{n}\to \alpha$, implying that the posterior density $f^{\left(n\right)}\left(p|x\right)$ of r.v. P concentrates in a neighbourhood of a certain point α as the sample size n grows.

The goal of the experimental design is twofold: (i) to maximise the number of responses in the experimental sample and (ii) to collect enough information on both treatment arms to make a statistically significant conclusion regarding the relative efficacy for the treatment arms. In such experiment, the quantitative measure of an information gain is provided by a weighted information measure (Mozgunov and Jaki, 2019). Let γ be the target response probability defined by a clinician. To emphasise the desirable values of the response probability (a neighbourhood of the target γ), the weight function in a Beta form can be used

(8) $$\phi_{\kappa }^{\left(n\right)}\left(p\right)=\overset{¯}{\Lambda }\left(\gamma ,x,n,\upsilon ,\beta ,\kappa \right)p^{\gamma n^{\kappa }}{\left(1-p\right)}^{\left(1-\gamma \right)n^{\kappa }}$$

where κ is the sensitivity parameter and $\overset{¯}{\Lambda }$ is a constant satisfying the normalisation condition

(9) $$\int_{R}\phi_{\kappa }^{\left(n\right)}f^{\left(n\right)}dp=1.$$

The weight function emphasises an interest in a certain area of outcomes, e.g. in this article that is a neighbourhood of a target efficacy probability γ. In other words, a greater “weight” is assigned to the information obtained about the arm with characteristics close to the desired target. To preserve the asymptotically unbiased estimation of the probability, the weight function is restricted to satisfy

$$\underset{n\to \infty }{\lim}\int_{0}^{1}p\phi_{\kappa }^{\left(n\right)}f^{\left(n\right)}dp=\alpha .$$

Then,

$$\int_{0}^{1}p\phi_{\kappa }^{\left(n\right)}f^{\left(n\right)}dp=\frac{x+\upsilon +\gamma n^{\kappa }+1}{n+\beta +n^{\kappa }+2}\overset{n\to \infty }{⟶}\alpha ,\;\kappa \in \left(0,1\right)$$

for the weight function (8). So, the condition of the unbiased estimation holds for 0<κ<1.

Following the general theory (MacKay, 2003), the information gain of the experiment is measured by the difference between the differential entropy and the weighted differential entropy. The difference between the entropies can also be considered as an average amount of the additional statistical information required when considering the context-dependent estimation problem instead of the “standard” one.

Following the conventional information gain approach, one would like to make a decision which maximises the statistical information in the experiment. It was already shown in Mozgunov and Jaki, 2019, Mozgunov and Jaki, 2020 that the leading term in the asymptotic difference between the entropies (as the size of experimental sample n→∞) can be used to govern the decision-making process during the experiment. Precisely, Mozgunov and Jaki (2020) obtained that the leading term of the difference between the weighted and standard Shannon differential entropy for a r.v. with density (7) and the weight function (8) takes the form

$$\frac{{\left(\alpha -\gamma \right)}^{2}}{2\alpha \left(1-\alpha \right)}{\left(n+\beta +2\right)}^{2\kappa -1}.$$

Below, we elaborate the same idea using other weighted generalisation of the well-established information measures, namely the Renyi, Tsallis and Fisher informations.

The following theorem provides an insight on why the difference of the standard and weighted information measures is a reliable criterion to govern the selection in the considered type of experiments.

Theorem 1 *Let* $\phi_{\kappa }^{\left(n\right)}$ *be the weight function given in* (8)*,* 0<κ<1*, and* $\lim_{n\to \infty }\frac{x}{n}=\alpha$*. Consider a r.v.* $Z_{x}^{\left(n\right)}$ *with PDF* (7)*. Then the following limits hold for the difference in the weighted and standard differential entropies of r.v.* $Z_{x}^{\left(n\right)}$ *for:*

*(i)* *the Renyi entropy*
  (10) $$\underset{n\to \infty }{\lim}\left[H_{\nu }^{\phi_{\kappa }}\left(f_{x}^{\left(n\right)}\right)-H_{\nu }\left(f_{x}^{\left(n\right)}\right)-\frac{1}{1-\nu }\omega \left(\nu ,\alpha ,\kappa ,n,\gamma \right)\right]=0$$
  $$\text{where}\;\omega \left(\nu ,\alpha ,\kappa ,n,\gamma \right)=\overset{\left\lfloor \frac{\kappa }{1-\kappa }\right\rfloor }{\underset{i=1}{\sum }}{\left(-1\right)}^{i-1}\frac{1}{i\left(i+1\right)}\left(\gamma^{i+1}\frac{1-\nu^{i}}{{\left(\nu \alpha \right)}^{i}}+\;{\left(1-\gamma \right)}^{i+1}\frac{1-\nu^{i}}{{\left(\nu \left(1-\alpha \right)\right)}^{i}}\;+\;\frac{\nu^{i}-1}{\nu^{i}}\right)n^{\left(i+1\right)\kappa -i};$$
*(ii)* *the Tsallis entropy*
  (11) $$\underset{n\to \infty }{\lim}\left[T_{q}^{\phi_{\kappa }}\left(f_{x}^{\left(n\right)}\right)-\exp\left\{\omega \left(q,\alpha ,\kappa ,n,\gamma \right)\right\}T_{q}\left(f_{x}^{\left(n\right)}\right)\right]=0;$$
*(iii)* *the Fisher information*
  (12) $$\underset{n\to \infty }{\lim}\left[I^{\phi_{\kappa }}\left(f_{x}^{\left(n\right)}\right)\;-\;I\left(f_{x}^{\left(n\right)}\right)\;\;-\underset{m_{1},m_{2}\in N:\;m_{1}+m_{2}\leq \left\lfloor \frac{2}{1-\kappa }\right\rfloor }{\sum }\frac{{\left(-1\right)}^{m_{1}+m_{2}-2}}{m_{1}m_{2}}\;\left({\left(\frac{\gamma }{\alpha }\right)}^{m_{1}}-{\left(\frac{1-\gamma }{1-\alpha }\right)}^{m_{1}}\right)\left({\left(\frac{\gamma }{\alpha }\right)}^{m_{2}}-{\left(\frac{1-\gamma }{1-\alpha }\right)}^{m_{2}}\right)n^{\left(m_{1}+m_{2}\right)\kappa -\left(m_{1}+m_{2}\right)+2}\;\;-2\overset{\left\lfloor \frac{\kappa }{1-\kappa }\right\rfloor }{\underset{m=1}{\sum }}\left({\left(\frac{\gamma }{\alpha }\right)}^{m}-{\left(\frac{1-\gamma }{1-\alpha }\right)}^{m}\right)\left(\frac{1-2\gamma }{2\alpha \left(1-\alpha \right)}\right)n^{\left(m+1\right)\kappa -m}\right]=0.$$

Proof The proof is given in Supplementary Materials.

Theorem 1 implies that for any entropy measure the asymptotic information gain is always non-positive and achieves the maximum value 0 at point α=γ. Thus, when maximising the information gain, one tends to collect more information about the arm corresponding to the response probability α closest to the target γ. Therefore, we propose to use them to govern the selection in the considered type of trials.

### 2.2 Criteria for the arm selection

Below, two types of designs which are based on the maximum information gain principle are proposed: (i) one is based on the criteria constructed using only the leading term of the asymptotic expansion, which, following the reasoning proposed by Mozgunov and Jaki (2020), can be easily interpreted and communicated to the clinical team, (ii) another uses the exact expression of the difference between the information measures.

For the first type of designs, using the leading term of the asymptotic expansion for difference between the weighted and standard Shannon (13) and Fisher (14) informations results in the following “asymptotic” criteria for the treatment selection

(13) $${\overset{¯}{\delta }}_{S}^{\left(\kappa \right)}\left(\gamma ,\alpha ,n,\beta \right)=\frac{{\left(\alpha -\gamma \right)}^{2}}{2\alpha \left(1-\alpha \right)}{\left(n+\beta +2\right)}^{2\kappa -1},\;\;\kappa \in \left[0.5,1\right)$$

and

(14) $${\overset{¯}{\delta }}_{F}^{\left(\kappa \right)}\left(\gamma ,\alpha ,n,\beta \right)=\frac{{\left(\alpha -\gamma \right)}^{2}}{\alpha^{2}{\left(1-\alpha \right)}^{2}}{\left(n+\beta +2\right)}^{2\kappa },\;\;\kappa \in \left(0,1\right),$$

respectively. Since (β+2) are the prior observations, the term (n+β+2) plays the role of “total” number of actual (n) and prior observations. Note that the leading terms for the Renyi and Tsallis entropies are not considered as they are monotonic transformations of the leading term of the difference for the Shannon entropies (13).

The numerator of the first term in both criteria given number of responses is a squared distance between the unknown probability α and the target probability γ. The denominator in (13) is a variance of Bernoulli r.v., while the denominator in (14) is a corresponding Wedderburn variance (McCullagh and Nelder, 1989). Therefore, the criteria can be considered as normalised distances between α and γ or, in other words, standardised statistics. Indeed, for known α and κ=0.75, (13) equals the squared t-statistics for the proportion. Noteworthy, the quantity which is well-established in statistics appeared independently using the information-theoretical argument. The second term depending on the number of patients n and the parameter κ, reflects the penalty on the number of patients assigned to the arm. To estimate the criteria above, the mean of posterior Beta distribution $\overset{ˆ}{\alpha }=\frac{x+\upsilon +1}{n+\beta +2}$ is “plugged-in” for α.

Since the asymptotic criteria ${\overset{¯}{\delta }}_{S}$ and ${\overset{¯}{\delta }}_{F}$ represent the amount of information obtained on each of the arms, to maximise the amount of information from the experiment, the arm with smaller value of $\overset{¯}{\delta }$ should be chosen. The first term in these measures, namely, the normalised squared difference of the target and the estimated probability of response, explicitly represents the interest in “exploitation”. The closer the estimated response probability is to the target one, the smaller the criterion will be and the design would tend to choose the corresponding arm. The second term, which shows positive dependence on the number of observations n and the build-in parameter κ (that is specified prior to the trial) represents the interest in “exploration”. In particular, for the AS the range of values of κ∈[0.5,1) shows all possible functional dependences on (n) between nth root to linear relationship, while for the AF the range of values of κ∈[0,1) shows all possible functional dependences on (n) between nth root to quadratic relationship. Hence, the higher the parameter κ the greater the investigator’s interest in the exploration and, consequently, the greater the penalty for each of new observation on the same arm. The guidance on the selection of the parameter κ is given in Section 3.

For the second type of designs, the differences in the weighted and standard Renyi (15), Tsallis (16), Fisher (17) and Shannon (18) differential entropies are used in order to build up the following “exact” criteria for the decision rules

(15) $$\delta_{R}^{\left(\kappa \right)}\left(\gamma ,n,x,\beta ,\upsilon \right)=H_{\nu }^{\phi_{\kappa }}\left(f_{x}^{\left(n\right)}\right)-H_{\nu }\left(f_{x}^{\left(n\right)}\right),$$

(16) $$\delta_{T}^{\left(\kappa \right)}\left(\gamma ,n,x,\beta ,\upsilon \right)=T_{\nu }^{\phi_{\kappa }}\left(f_{x}^{\left(n\right)}\right)-T_{\nu }\left(f_{x}^{\left(n\right)}\right),$$

(17) $$\delta_{F}^{\left(\kappa \right)}\left(\gamma ,n,x,\beta ,\upsilon \right)=I^{\phi_{\kappa }}\left(f_{x}^{\left(n\right)}\right)-I\left(f_{x}^{\left(n\right)}\right),$$

(18) $$\delta_{S}^{\left(\kappa \right)}\left(\gamma ,n,x,\beta ,\upsilon \right)=h^{\phi_{\kappa }}\left(f_{x}^{\left(n\right)}\right)-h\left(f_{x}^{\left(n\right)}\right),$$

respectively.

### 2.3 Design

As each of the information-theoretical criterion already carries an information on the uncertainty regarding the treatment arms, the following “best intention treatment” type of designs is proposed based on the derived information gain measures.

Consider k alternative treatment arms $\left\{A_{1},\ldots ,A_{k}\right\}$. Denote by $\alpha_{j}$, $\upsilon_{j}$, $\beta_{j}$, $n_{j}$ and ${\overset{ˆ}{\alpha }}_{j}$ parameters for the arm $A_{j}$. For asymptotic designs the experiment starts with the arm that minimises either the criterion (13) or the criterion (14) based on the prior distribution: $\inf_{j\in \left\{1,\ldots ,k\right\}}\left\{{\overset{¯}{\delta }}_{i}^{\left(\kappa \right)}\left(\gamma ,{\overset{ˆ}{\alpha }}_{j},0,\beta_{j}\right)\right\}$, i∈{S,F}, ${\overset{ˆ}{\alpha }}_{j}=\frac{\upsilon_{j}+1}{\beta_{j}+2}$.

Once the outcomes for the previous $n=n_{1}+\cdots +n_{k}$ patients are observed, the total number of responses is updated, and the plug-in estimator ${\overset{ˆ}{\alpha }}_{j}=\frac{x_{n}+\upsilon_{j}+1}{n_{j}+\beta_{j}+2}$ and the information gains ${\overset{¯}{\delta }}_{i}^{\left(\kappa \right)}\left(\gamma ,{\overset{ˆ}{\alpha }}_{j},n_{j},\beta_{j}\right)$ are recomputed. The next patient is assigned to the treatment arm corresponding to the minimum values of the criterion $\inf_{j\in \left\{1,\ldots ,k\right\}}\left\{{\overset{¯}{\delta }}_{i}^{\left(\kappa \right)}\left(\gamma ,{\overset{ˆ}{\alpha }}_{j},n_{j},\beta_{j}\right)\right\}$.

This procedure repeats until the total number of observations $N=n_{1}+\cdots +n_{k}$ is attained. For the final treatment recommendation, in order to eliminate the weight function influence, the parameter κ is set on the level that minimises the leading term in the asymptotic. Namely, κ is set to $\kappa_{f}=\frac{1}{2}$ for the Shannon criterion and $\kappa_{f}\approx 0$ for the Fisher criterion (Mozgunov and Jaki, 2020). The target arm is then defined in the same manner: $\inf_{j\in \left\{1,\ldots ,k\right\}}\left\{{\overset{¯}{\delta }}_{i}^{\left(\kappa_{f}\right)}\left(\gamma ,{\overset{ˆ}{\alpha }}_{j},\beta_{j},\upsilon_{j}\right)\right\}$ where plug-in estimator ${\overset{ˆ}{\alpha }}_{j}$ is computed with $x_{j}$ and $n_{j}$, the total number of positive responses and observations for the arm $A_{j}$, respectively.

The algorithm for the exact criteria mimics the one described above. The experiment starts with the arm that minimises one of the quantities (15)–(18), depending on the information measure used, based on prior distribution: $\inf_{j\in \left\{1,\ldots ,k\right\}}\left\{\delta_{i}^{\left(\kappa \right)}\left(\gamma ,0,0,\beta_{j},\upsilon_{j}\right)\right\}$, i∈{R,T,S,F}.

Once the outcomes for the previous $n=n_{1}+\cdots +n_{k}$ patients are observed and the information gain $\delta_{i}^{\left(\kappa \right)}\left(\gamma ,n_{j},x_{j},\beta_{j},\upsilon_{j}\right)$ is recomputed with an updated number of responses, the target arm for the next patient is being chosen by the rule: $\inf_{j\in \left\{1,\ldots ,k\right\}}\left\{{\overset{¯}{\delta }}_{i}^{\left(\kappa \right)}\left(\gamma ,n_{j},x_{j},\beta_{j},\upsilon_{j}\right)\right\}$. The procedure repeats until the total number of N observations is attained. At the end of the experiment as for the asymptotic criteria, the target arm is defined with $\kappa_{f}$ which minimises leading term of the asymptotic: $\kappa_{f}=\frac{1}{2}$ for the Renyi, Tsallis and Shannon criteria and $\kappa_{f}\approx 0$ for the Fisher criterion.

The proposed algorithms are myopic in a sense that on each step given the collected information at each step the decision is made only with regard to the one next subject (Hu and Rosenberger, 2006).

## 3 Comparison of designs based on different context-dependent measures

Below, we consider how different information measures influence the operating characteristics of the design. First, the setting of the proposed simulation study is defined. Further, the calibration of design parameters for each criterion is described. Then it is studied whether the use of asymptotic or exact criteria results in differences in the designs’ properties and the balance between the trial objectives. Afterwards, a comparison of the designs with different penalty parameters and different information measures is performed.

### 3.1 Setting

To study the characteristics of the design, we consider the clinical setting in a rare disease trial with two treatments arms and binary efficacy responses which was originally proposed in Williamson et al. (2017).

Following the original notations, probabilities of response for arm A and arm B are denoted by $\theta_{a}$ and $\theta_{b}$, respectively. The total sample size is fixed to be N=75. The target response probability is set to be γ=0.999 indicating the interest in outcomes with high efficacy and that was found to result in good operating characteristics (Mozgunov and Jaki, 2020).

Two main objectives of the experiment are to maximise the number of responses in the experimental sample and to make a statistically significant conclusion regarding the relative efficacy for the treatment arms. For the second goal, a comparative hypothesis $H_{0}:\theta_{a}=\theta_{b}$ is tested at the end of the experiment against the two-sided alternative. In order to test $H_{0}$, Fisher’s exact test (Upton, 1992) is used. The probability $\theta_{b}=0.5$ was fixed and $\theta_{a}$ obtained each value from the set $\Theta_{a}=\left\{0.1,0.2,\ldots ,0.9\right\}$. A combination $\left(\theta_{a};\theta_{b}\right)$ will be referred to as a scenario. Furthermore, in a phase II settings, a question of correct arm selection could be also of a major interest to investigators. These trials’ objectives are translated into the following operating characteristics to be studied:

1. **Type I error rate.** The proportion of times $H_{0}$ is incorrectly rejected under scenario $\theta_{a}=\theta_{b}=0.5$. The type I error rate is required to be controlled under 10%.
2. **Power.** The proportion of times $H_{0}$ is correctly rejected under scenarios $\theta_{a}\neq \theta_{b}$.
3. **Proportion of correctly allocated patients (PCA).** The proportion of patients on a superior treatment.
4. **Probability of correct selections (PCS).** The proportion of times when the truly superior arm was correctly recommended by the design.

These are the characteristics in terms of which the designs will be evaluated.

### 3.2 Calibration

In this section, we provide details on the calibration of the parameters of prior distribution and additional parameters q for the Tsallis and ν for the Renyi criteria in an extensive simulations study. Computer simulations for each scenario involved 10,000 trial replications. The main goal of the calibration procedure is to find the values of the parameters of prior distribution such that the type I error under the scenario of equally good treatments is controlled at 10% level for all values of κ∈{0.1,0.11,…,0.9}. After that, among the designs with the calibrated parameters, we search for the values q and ν which allows for an advantage in terms of power or PCA. The main purpose of this part of calibration is to find the design parameters controlling the type I error for all values of the parameter κ that will be later used to balance the “learn versus earn” trade-off.

To describe the prior distribution, we introduce the quantities E>0 and η∈(0,1) called the strength of prior and prior probability, respectively. Interpreting these parameters, the prior distribution has a strength of prior (effective sample size of) E with prior probability η implying that it takes the form: B(E×η,E−E×η). To satisfy the principle of clinical equipoise (Djulbegovic, 2009) the same prior probability η=0.99 and strength of prior E were set for each arm. Note that for any E such choice reflects no prior knowledge about which arm is superior, and each treatment is considered as highly efficacious (with the response probability of 0.99) until data suggests otherwise.

The following effect of E on the operating characteristics can be observed: higher values of E correspond to a better performance in terms of power, but a worse performance in terms of PCA. For larger values of E, in order to stop mistakenly considering one arm as highly efficacious, more information supporting the evidence that the arm is inferior is required, thus, more observations will be made on a less efficacious treatment. Therefore, one would prefer to keep E small. At the same time, it was found that for small values of E≤10, the type I error was not controlled for all values of the penalty parameter κ. To ensure the control of type I error at the desirable level, the adjusted Fisher test with different cut-off points δ was adopted. This cut-off point for the p-value was included as part of the calibration procedure.

The calibration of E was performed separately for each information measure. For all values of the cut-off parameter δ∈{0.08,0.085,…,0.1}, the smallest value of E∈{1,2,…,10} is chosen such that the type I error is controlled for all values of κ∈{0.01,0.02,…,0.99} on 10% level. After that among the pairs with minimal value of E, the one with the highest δ would be chosen. For all considered information gains, the pairs of the calibrated parameters are given in Table 1.

Afterwards, for the Tsallis and Renyi criteria, additional parameters q and ν were calibrated in a way that each of the designs yield an advantage in terms of power or PCA, other things being equal. During this step the following values of the parameters were chosen: q=0.35 for the Tsallis entropy and ν=0.75 for the Renyi entropy. The illustration of the effect of E and δ on type I error rate, finer details on the calibration procedure of all the parameters and the results for the Tsallis and Renyi criteria are given in Supplementary Materials.

**Table 1:** Calibrated values of E and δ for each entropy criteria, namely the Shannon (S), Fisher (F), Tsallis (T), Renyi (R).

	AS	AF	S	F	T	R
E	7	6	9	4	9	8
δ	0.09	0.085	0.085	0.09	0.09	0.09

A – asymptotic criteria, no letter – exact criteria.

In further evaluations, we focus on the designs based on Shannon, Fisher, and Tsallis criteria with the calibrated values of prior parameter E, cut-off parameter δ and additional parameter q. The designs based on the Renyi criterion will not be considered further as no qualitative differences were found between the designs based on Tsallis and Renyi entropy criteria with calibrated values of q and ν.

### 3.3 The effect of the penalty parameter κ on operating characteristics

Now, when the rest of the parameters are chosen such that the type I error is controlled for all values of κ, we investigate how the choice of penalty parameter κ (the core component of the proposed information measures allowing to tackle the Power-PCA trade-off) influences the operating characteristics. For convenience, the following notation will be used: as before, AF, F, AS, S,T,R refer to the corresponding entropy criteria, and 0.1,0.2,…,0.9 – to the value of κ, e.g. AF0.3 refers to the design based on asymptotic Fisher criterion with κ=0.3.

**Fig. 1:**
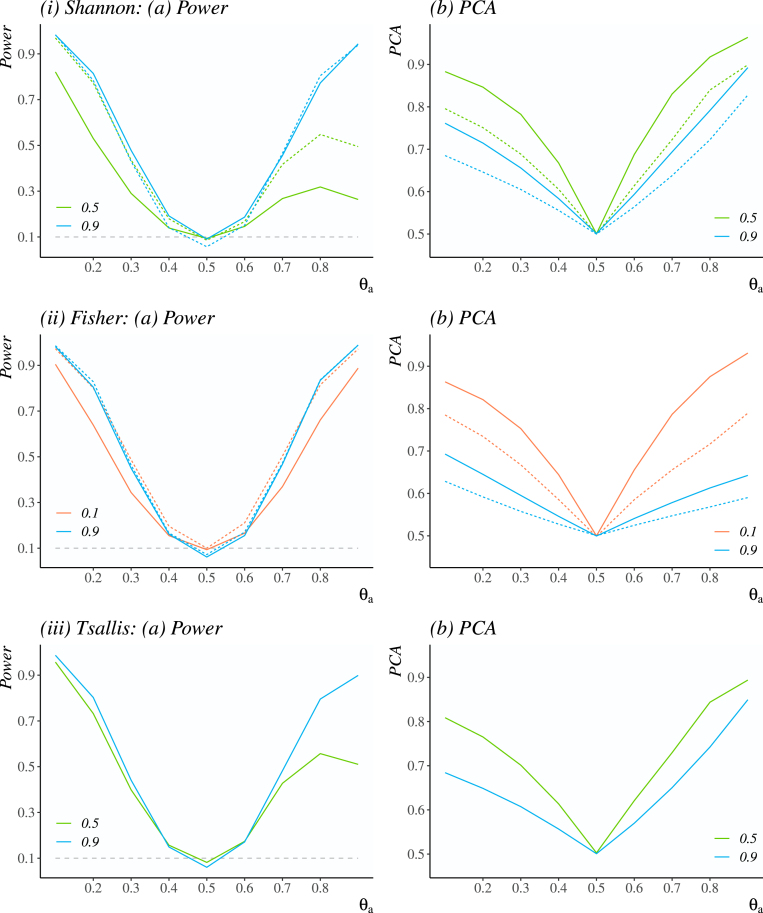
Power and PCA for the Shannon, Fisher and Tsallis criteria with different values of κ: κ=0.1 (red line), κ=0.5 (green line), κ=0.9 (blue line); the asymptotic criteria AS, AF, and T are denoted by solid lines, and the exact criteria S and F by the dashed line.(For interpretation of the references to colour in this figure legend, the reader is referred to the web version of this article.)

Fig. 1 illustrates power and PCA under various scenarios for the following values of the penalisation parameter: for the S and AS κ∈{0.5,0.9}, for the F and AF κ∈{0.1,0.9} and for the T κ∈{0.5,0.9}. Choosing these values of κ allows for an exploration of the designs corresponding to different balances between power and PCA.

It is found that, in general, greater values of κ correspond to a higher power. It follows that greater values of κ require an increased level of confidence that the selected arm is superior, which result in more frequent switching between arms and hence a more even allocation of patients. Conversely, smaller values of κ result in less frequent switching between arms and hence lead to a higher PCA as the designs tend to hone in on the more superior arm. Therefore, when interpreting the parameter κ, it should be noted that under the proposed design, a particular level of exploration is achieved via tuning of κ that is fixed prior to the trial. In fact, this tuning procedure can serve as a communication of the effect of κ on the properties of designs and should be used to inform the value of κ to be used in an actual trial.

The effect of κ on power and PCA is more prominent for scenarios that are further away from the one with equal probability of response $\theta_{a}=\theta_{b}=0.5$, e.g. between AF0.1 and AF0.9 in terms of PCA the highest difference of 8.8% is seen for scenarios with $\theta_{a}=0.9$, while for $\theta_{a}=0.4$ the difference of 2.7% is the lowest.

In addition, some designs are “symmetrical” with respect to the scenario $\theta_{a}=\theta_{b}=0.5$ in a way that under scenarios with $\theta_{a}\in \left\{0.1,\ldots ,0.4\right\}$ and $\theta_{a}\in \left\{0.9,\ldots ,0.6\right\}$ the operating characteristics are nearly the same, e.g. for AF0.9 in terms of power mean difference between the scenarios equally distanced from $\theta_{a}=\theta_{b}=0.5$ is of 1.2%. Conversely, the AS0.5 design is highly “asymmetrical” in terms of power with the highest difference of 55.7% between scenarios with $\theta_{a}=0.1$ and $\theta_{a}=0.9$. The difference in terms of PCA for that design is also slightly growing the further the scenarios are from being equal, with the highest difference of 8.1% between scenarios $\theta_{a}=0.1$ and $\theta_{a}=0.9$.

This effect is due to the form of the chosen weight function (8), and specifically, the parameter κ. Under scenarios with $\theta_{a}\geq 0.6$, the true response probability of arm A becomes closer to γ and under lower value of κ, most patients are assigned to the superior treatment arm A. For instance, in the AS0.5 design with the scenario $\theta_{a}=0.9$ with the probability of 83.7% 5 or less patients were assigned to treatment arm B (see Supplementary Materials for further details). Therefore, there might not be enough information to obtain statistically significant results. Note, that S0.5 and T0.5 were found to be highly asymmetrical in terms of power for the same reason.

### 3.4 Comparison of asymptotic and exact criteria

Further, we investigate whether the designs based on the criteria constructed using the leading term of asymptotic expression only differ from the exact entropy criteria in terms of the operating characteristics of interest. Consider the designs with κ∈{0.5,0.9} for the AS and S criteria and with κ∈{0.1,0.9} for the AF and F criteria. The operating characteristics for these designs are given in Fig. 1, Panel (i) and Panel (ii), respectively.

For the Shannon criterion, in terms of PCA, the use of asymptotic criteria leads to an increase around 6.2%–10.8% among non-null scenarios $\Theta_{a}^{\prime }$ for κ=0.5, and around 1.6%–6.4% for κ=0.9, in comparison to the exact criteria based designs with the same value of κ, whereas for the Fisher criterion the increase is around 5.9%–15.9% for κ=0.1, and around 1.6%–6.4% for κ=0.9.

In terms of power, for the Shannon criteria the use of asymptotic criteria leads to a loss around 2.1%–24.2% in comparison to the exact criteria for κ=0.5. However, for κ=0.9 the asymptotic criteria allows for a minor increase in terms of power with its maximum value of 5.1% for $\theta_{a}=0.4$ and 1.6% on average. For the Fisher criteria with κ=0.1, the use of asymptotic criteria leads to an average loss of around 4.1%–16.2%, whereas for κ=0.9 the exact criteria still outperforms asymptotic, though the difference is smaller, around 0.1%–2.4%. Considering the analysis for all values of κ, for κ≥0.3 the exact criteria still outperforms asymptotic on average, though the difference of <1% is negligible.

This result implies that choosing the asymptotic criteria over the exact one has the similar effect on PCA as lowering the penalty parameter κ as for the asymptotic criteria information gains are smaller than for the exact criteria since only the leading term is used, while by definition information gains depend positively on κ. Overall, under the most scenarios, the choice of asymptotic criteria results in noticeable increases in the PCA while resulting in marginal losses in power. If a balance provided by the exact criterion is thought to be more favourable, it could be achieved via increasing κ for asymptotic criterion. Finally, being mindful of the communication of the measures, the asymptotic criteria have a more intuitive interpretation. Following the rule “the simpler, the better”, for both entropy measures asymptotic criteria are preferred to the exact criteria.

### 3.5 Tuning of the penalty parameter κ

After the investigation of the effect of the penalty parameter κ on operating characteristics within each of the entropy criteria separately, the number of designs to consider is narrowed down to the AS and T with κ∈{0.5,…,0.9}, and AF with κ∈{0.1,…,0.9}. Although the direction of effect of κ for these designs is the same, the size of this effect varies depending on which entropy criterion the design is based on. Previously, we focused on a pairwise comparison of the designs with several values of κ in terms of their average performance. Below, we provide the guidance on how to select among the designs with various values of κ such that the desirable trade-off between power and PCA is achieved.

The concept of finding a balance between power and PCA can be subjective and depends on the investigator’s preference on the power and PCA. Besides, the “balance” itself can be considered unethical, i.e. patients are sacrificed to gain power. Thus, any formal criterion used to select the design with the “best” power-PCA balance should be viewed purely as a metric which allows for a consistent comparison among the competing approaches with respect to operating characteristics of interest.

At the first stage of our evaluations, we consider the measures of average performance across non-null scenarios $\Theta_{a}^{\prime }$. As in actual trials the true probabilities of response $\theta_{a}$ and $\theta_{b}$ are unknown, the averaging across scenarios implies that each of these scenarios is deemed equally likely *à priori*. Therefore, one would be looking for the design that under this prior belief provides a good balance in the operating characteristics. To compare different approaches among each other their average performances should be standardised. To achieve this we consider an average performance in terms of power and PCA relative to the conventional fixed randomisation (FR) design, which randomises patients to treatment arms A or B with the equal fixed probability. In terms of both operating characteristics the FR provides two extremes: high statistical power, but a low PCA, fixed at 0.5, $\forall i\in \Theta_{a}$. Formally, consider average percentage performance of a design X over the set of scenarios $\Theta_{a}^{\prime }$ in terms of power relative to the FR

(19) $${\bar{\psi }}_{X}≔\frac{100}{\left|\Theta_{a}^{\prime }\right|}\underset{i\in \Theta_{a}^{\prime }}{\sum }\frac{\psi_{X,i}-\psi_{FR,i}}{\psi_{FR,i}}\;,$$

and similarly defined average percentage performance in terms of PCA

(20) $${\bar{\phi }}_{X}≔\frac{100}{\left|\Theta_{a}^{\prime }\right|}\underset{i\in \Theta_{a}^{\prime }}{\sum }\frac{\phi_{X,i}-\phi_{FR,i}}{\phi_{FR,i}}\;$$

where $\psi_{X,i}$, $\phi_{X,i}$ are power and PCA for design X in a scenario i, respectively. An average performance of any design X in comparison to the FR in terms of power ${\overset{¯}{\psi }}_{X}$ is expected to be negative and will be further referred as “an average percentage loss in power”. Conversely, an average performance in terms of PCA ${\overset{¯}{\phi }}_{X}$ for any design will be positive relative to FR, therefore, will be referred as “an average percentage gain in PCA”. It is important to note that such aggregation metrics might hide some unexpected behaviour under individual scenarios, for example, the asymmetrical performance of AS05 discussed in Section 3.3. Therefore, these metrics should not be the single measure to base the conclusion on. Instead, it is used to provide guidance on the parameter κ selection. To uncover such potential behaviour under some scenarios, once the parameters are chosen, we will consider individual scenarios when comparing the proposed designs to alternative approaches.

**Fig. 2:**
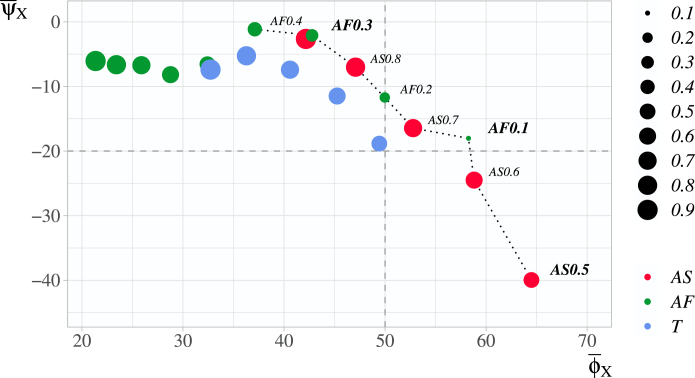
An average percentage power loss ${\overset{¯}{\psi }}_{i}$ vs an average percentage PCA gain ${\overset{¯}{\phi }}_{i}$ for design X in comparison to the FR approach for the AS (red dots), AF (green dots). The size of dots corresponds to the value of κ: smallest dot, κ=0.1; largest dot, κ=0.9. Dotted line connects the designs that cannot be improved in terms of one characteristic while not worsening in another. The designs favouring the power are represented by the dots which lie above a horizontal dashed line; the designs favouring the PCA — to the right of a vertical dashed line.(For interpretation of the references to colour in this figure legend, the reader is referred to the web version of this article.)

Fig. 2 shows an average performance of all competing approaches relative to the FR design, as defined in (19), (20), with each dot representing one design from the set of competing approaches. For each design X the dots to its right correspond to the designs which outperform X in terms of PCA, while the dots above correspond to the designs which outperform X in terms of power.

For all entropy criteria, the same effect as found before can be observed — lowering of κ leads to an increase of an average percentage gain in terms of PCA. In terms of power lowering of κ results either in an increase of an average loss of power in comparison to FR (for AS with all values of κ, for the AF with κ<0.5, for T with 0.2<κ<0.9), or no noticeable difference, as for AF with κ≥0.5.

However, in terms of average percentage performance relative to the FR the difference between the designs is observable with the dots representing AF0.4 and AF0.3 lying higher than the AF with κ≥0.5. This is caused by the fact that, in terms of power, the FR outperforms the AF with κ∉{0.3,0.4} for all scenarios $\theta_{a}$, whereas for $\theta_{a}$ close to 0.5 the FR is outperformed by both AF0.4 and AF0.3 (e.g. in case of AF0.3, as can be observed in Fig. 4, by 1.6% and 2.0% for $\theta_{a}\in \left\{0.4,0.6\right\}$, respectively). This advantage in terms of power for scenarios with $\theta_{a}$ close to 0.5 is also seen for T0.8, T0.7, which explains why these dots lie higher than the dot corresponding to T0.9 in Fig. 2.

The designs which cannot be improved in terms of one characteristic while not worsening in another, correspond to the dots, for which no dots lie on the top right. These designs, namely the AS with all values of κ and the AF with κ<0.5, are represented in Fig. 2 by the dots connected with a dotted line. Note, that this list does not include the designs based on the Tsallis criterion, as well as the Renyi criterion (see Supplementary Materials). These designs provide a better power-PCA balance in comparison to the rest of the comparators. However, each of them represents different balances in a way that AF0.1 corresponds to the design with a balance shifted towards power, while AS0.5 corresponds to the design with a balance shifted towards PCA. We will treat the designs with mean percentage power loss <20% as favouring the power and the designs with mean percentage PCA gain >50% as favouring the PCA. The values for these boundaries are calculated as a rounded mean between the best and the worst performance in terms of each of the operating characteristics among the designs with better power-PCA balances. Based on the defined intervals the entropy based designs that cannot be improved upon are divided into three groups:

• AF0.4, AF0.3 and AS0.8 with a balance “shifted” towards power;
• AS0.7 and AF0.1 with an “intermediary” balance;
• AS0.5 and AS0.6 with a balance “shifted” towards PCA.

To choose one design within each group we use a linear trade-off function between an average percentage power loss and average percentage PCA gain

(21) $$B_{XY}=\left|\frac{{\bar{\psi }}_{X}-{\bar{\psi }}_{Y}}{{\bar{\phi }}_{X}-{\bar{\phi }}_{Y}}\right|$$

for neighbouring designs X and Y. $B_{XY}$ shows how many percents of power are given in return for a 1% gain in terms of PCA on average, $B_{XY}^{-1}$ shows how many percents of PCA are given in return for a 1% gain in terms of power on average.

Between the approaches representing the balance shifted towards power, choosing the AF0.3 over AF0.4 corresponds to $B_{1,2}^{-1}=5.9\text{\%}$ of average gain of PCA in return for 1% loss of power, whereas choosing the AS0.8 over AF0.3 corresponds to $B_{2,3}^{-1}=0.88\text{\%}<1\text{\%}$ of average gain of PCA in return for 1% loss of power. Therefore, if power is favoured over PCA, AF0.3 should be chosen as a design with desired characteristics.

Further, consider the approaches representing the balance shifted towards PCA. Choosing AS0.5 over AS0.6 results in $B_{7,8}=2.7\text{\%}$ of average gain of power in return for 1% loss of PCA. However, the analysis of the effect of κ on scenario-by-scenario basis has shown that the significant drawback in power of AS0.5 in comparison to the FR is caused mostly by scenarios with $\theta_{a}>0.7$ (see Fig. 1); therefore, if we are favouring PCA over power AS0.5 should be chosen between these two.

Finally, between the approaches representing the intermediary balance, choosing the AF0.1 over AS0.7 results in $B_{5,6}^{-1}=3.5\text{\%}$ of average gain of PCA in return for 1% loss of power. Note, that the AF0.1 is also preferred over AS0.6, which represents a design balanced towards PCA, since it results in $B_{6,7}=11.6\text{\%}$ of average gain of power in return for 1% loss of PCA.

In the next section, the proposed designs with the parameter values that were found to induce the superior power-PCA balance, namely AF0.1,AF0.3 and AS0.5, will be compared to two alternative approaches.

## 4 Comparison to alternative approaches

Below, we investigate whether the proposed design based on the information gain criteria with the weighted information measures can offer further advantages compared to these response-adaptive designs in a comprehensive simulation study with the same trial setting as above.

### 4.1 Alternative approaches

We will consider the fixed equal randomisation design and optimal dynamic programming (DP) approach as two design options that allow to achieve the highest power and PCA, respectively. We also include the extension of the DP, optimal constrained randomised dynamic programming (CRDP) (Williamson et al., 2017) for a number of design parameter choices. The CRDP is selected as a flexible approach that can achieve various power-PCA balances, similarly to the proposed approach.

The CRDP design based on dynamic programming principles allows to take into account all possible future outcomes in the selection decisions. In the setting of the CRDP design, the prior information about the unknown parameters is used in conjunction with observed responses and the number of patients still to be enrolled in the trial to determine the optimal treatment allocation for every patient.

The CRPD design has built-in parameters, the randomisation parameter p and constraint parameter l, which provides some variability in choosing different types of power-PCA balances. Constraint parameter l ensures that each treatment arm always obtains at least l observations. Hence, l=0.5n corresponds to the fixed equal randomisation. Randomisation parameter 0.5≤p≤1 assigns a probability to the allocation rule at each stage, so that each of the treatments has a probability of at least 1−p of being allocated to each patient. In particular, p=0.5 correspond to the fixed equal randomisation, and p=1 corresponds to the dynamic programming approach (DP), and the values between result in balances of these two extremes. The choice for randomisation parameter, according to recommendations given in Williamson et al. (2017), is p=0.9. Similarly, values 0.10n<l<0.15n were argued to yield robust design characteristics.

To specify the values of the CRDP parameters for the comparison to the proposed entropy-based designs, we have evaluated CRDP using values of p∈{0.6,0.7,…,1} and l∈{0.05n,0.1n,…,0.5n}. The operating characteristics of the CRDP designs were computed in a simulation study with 10,000 trial replications. Based on additional simulations (provided in Supplementary Materials), we have focused on the CRDP design with p=0.9 in line with the original recommendation, since for all values of l it consistently gave an advantage in terms of PCA in return for an equally good performance in terms of power between the designs. For convenience, the following notation will be used: CRDP will refer to the comparator design name and 0.05,0.1,…,0.5 to the value of l∕n, e.g. CRDP0.1 refers to the CRDP design with p=0.9 and l=0.1n.

### 4.2 Numerical results

**Fig. 3:**
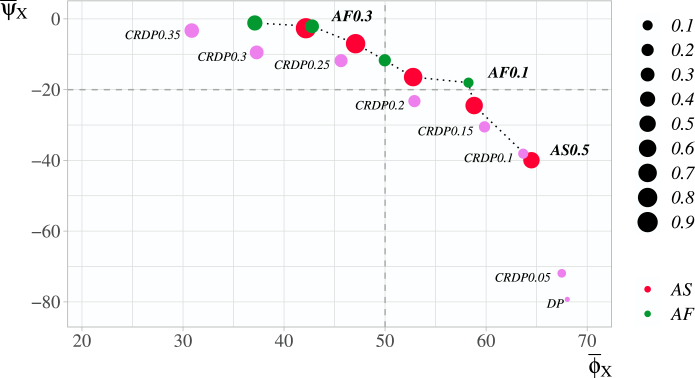
An average percentage power loss ${\overset{¯}{\psi }}_{i}$ vs an average percentage PCA gain ${\overset{¯}{\phi }}_{i}$ for winning entropy-based designs X and dynamic programming designs (CRDP and DP, purple dots) in comparison to the FR approach. The size of dots corresponds to the value of κ: smallest dot, κ=0.1; largest dot, κ=0.9. Dotted line connects the designs that cannot be improved in terms of one characteristic while not worsening in another. The designs favouring the power are represented by the dots which lie above a horizontal dashed line; the designs favouring the PCA — to the right of a vertical dashed line.

Fig. 3 presents the average performance of the CRDP and DP approaches relative to the FR, alongside with the three designs with tuned κ parameter representing different balances between power and PCA, namely the AF0.1, AF0.3 and AS0.5. Values ${\overset{¯}{\psi }}_{i}$ and ${\overset{¯}{\phi }}_{i}$ for the alternatives are also calculated via (19), (20), respectively.

For the CRDP designs with l∕n≥0.25 a balance is shifted towards power, since, relative to the FR, an average percentage loss of power is below 20%, and an average percentage gain in PCA is below 50%. On the contrary, for the CRPD with l∕n≤0.2, a balance is shifted towards the PCA.

The CRDP0.35 has the closest to AF0.3 performance in terms of power with around 1% loss in power on average, but noticeably lower average PCA with a difference of 11.9%. The CRDP0.1 performs similarly to AS0.5, while corresponding to a 5.4% gain in PCA and loss of 20.1% in power compared to AF0.1. The AS0.5 in comparison to the DP provides an average percentage increase in terms of power of 39.3% in exchange for an average percentage PCA loss of 3.5%.

**Fig. 4:**
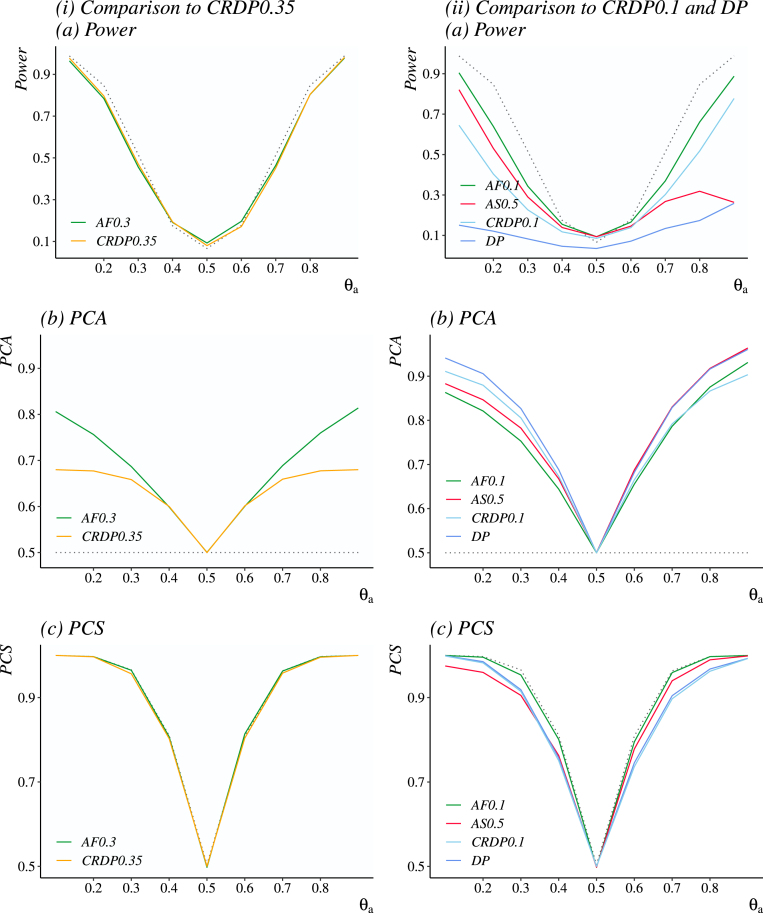
Comparison of the novel designs, namely AF0.1 (green line), AF0.3 (green line) and AS0.5 (red line), to the alternative approaches, namely CRDP0.35 (orange line), CRDP0.1 (light blue line), the DP (blue line), the FR (grey dotted line), in terms of power, PCA and PCS.(For interpretation of the references to colour in this figure legend, the reader is referred to the web version of this article.)

The scenario-by-scenario comparison of these designs in terms of Power and PCA is given in Fig. 4 (Panels (a) and (b)). As a researcher might be also interested in the objective of making a correct arm selection by the end of the trial, we will also consider the probability of correct selections (PCS) in our evaluation given in Fig. 4 (Panel (c)). The left-hand side subplots represent the AF0.3 alongside with the CRDP0.3, since they both provide a power-PCA balance shifted towards power, and the FR. The right-hand side subplots representing AF0.1 and AS0.5 alongside with the CRDP0.1 and DP, since these are constructed to achieve higher PCA, and AF0.1 provides an intermediary power-PCA balance. Important to note, that all the following comparisons were made for the calibrated values of the parameters.

Considering the designs with a balance shifted towards power, AF0.3 correspond to 6% higher PCA, on average, compared to CRDP0.35 under scenarios $\theta_{a}\notin \left\{0.4,0.6\right\}$, while performing similarly under $\theta_{a}\in \left\{0.4,0.6\right\}$ and resulting in nearly the same average power across all scenarios (with differences not exceeding 0.1% in individual scenarios). Comparing FR and AF0.3, the FR design corresponds to 2.7% higher power, on average, with the maximum difference across scenarios not exceeding 7.5% and AF0.3 outperforming the FR by 1.6% and 2% under scenarios $\theta_{a}\in \left\{0.4,0.6\right\}$. In terms of PCA, the average difference between FR and AF0.3 is of 21.4%. All of the designs favouring power result in nearly the same PCS.

Considering the design with an intermediary balance, AF0.1 outperforms CRDP0.1 in terms of power for all scenarios with an average difference of 12.5%. In terms of PCA, for the scenarios with $\theta_{a}<0.5$ it is outperformed by CRDP0.1 with an average of 4.7%, performs similarly under $\theta_{a}\in \left\{0.6,0.7,0.8\right\}$ with differences not exceeding 0.9% and corresponds to a gain of 2.8% for $\theta_{a}=0.9$. The AF0.1 outperforms CRDP0.1 in terms of PCS for all of the scenarios with an average difference of 3.8%.

Considering the designs shifted towards PCA, for scenarios with $\theta_{a}<0.7$ AS0.5 outperforms CRDP0.1 in terms of power with an average difference of 7.9%, while for scenarios with $\theta_{a}\geq 0.7$ AS0.5 is outperformed by the CRDP0.1 with a maximum difference reaching 51.4%. In terms of PCA, for all of the scenarios with $\theta_{a}<0.5$, AS0.5 is outperformed by both CRDP0.1 and DP, with the average difference of 2.2% and 4.6%, respectively. For all of the scenarios with $\theta_{a}>0.5$, the difference between the DP and AS0.5 is no more than 0.5%. In terms of PCS, AS0.5 is outperformed by both the DP and CRDP0.1 for the scenarios with $\theta_{a}<0.4$, and vice versa for $\theta_{a}\geq 0.4$, with the difference not exceeding 4.2%.

Overall, the proposed information-theoretic approach allows to tackle the problem of finding a desired trade-off explicitly with the parameter κ. If the researcher favours power over PCA, AF0.3 provides good operating characteristics among the comparators. If the researcher favours PCA over power, bearing in mind the problems with power for scenarios with $\theta_{a}\geq 0.7$, the AS0.5 design might be considered among the alternatives. However, if the advantage in power is more prominent than the drawback in PCA, then AF0.1 should be selected, since this design allows to increase the statistical power while providing substantial benefits in terms of the patients receiving the superior treatment.

## 5 Discussion

In this article, the recently proposed response-adaptive designs for Phase II clinical trials based on the context-dependent information measure (Mozgunov and Jaki, 2020) are extended to various information measures, namely the Renyi, Tsallis and Fisher informations. The new designs use the maximum information gain principle, which is measured via the difference between the weighted and standard Renyi, Tsallis, Shannon, and Fisher informations.

The asymptotic behaviour of the information gain is investigated for each information measure in order to construct an easily interpretable criteria for the arm selection. As a result, the asymptotic Fisher criterion was derived in addition to the asymptotic Shannon criterion proposed in Mozgunov and Jaki (2020). It was shown that using exact criteria brings no sufficient gain in terms of the power or PCA, and has a similar effect on operating characteristics as an increase in the penalty parameter κ. As the results, we recommend the asymptotic criteria for each information measure as these could be easier communicated compared to the exact formulae.

The designs based on the derived asymptotic information criteria were evaluated in the simulation study. Firstly, an extensive procedure of the design parameters was performed to ensure the control of the type I error. This consists of a large number of simulations under various designs parameters. Secondly, via tuning of the penalty parameter κ, it was found that the proposed design can attain various power-PCA balances. Again, the choice of κ was driven by extensive simulations to ensure favourable operating characteristics. At the same time, the calibration process itself could serve as a communication of the interpretation of κ and its influence on the trial design.

For the calibrated design parameters, in comparison to the alternative designs based on the dynamic programming approach, the proposed entropy-based approach can provide several design specifications to choose from, while improving on at least one of the characteristic of interest. The design based on Fisher Information under selected κ allows for a statistical power similar to the FR with noticeably higher PCA under all considered scenarios. Conversely, the design based on the Shannon criterion allows for an advantage in terms of power and PCS in comparison to the DP while resulting in a minor drop in the PCA.

While the two-arm setting has been considered only, there is an increasing interest in multi-arm trial and the performance studied in this work encourages further exploration of the design properties in multi-arm setting, which is the subject to future research. Furthermore, the fundamental assumptions of the proposed design (together with other response-adaptive alternatives) is that the patients’ outcomes are quickly observed. However, there are many setting in which there is a delay in evaluating the efficacy of response (Rosenberger, 1999). Recently, the methodology based on the approximated binomial likelihood approach (Lin et al., 2020, Lin and Yuan, 2020) was proposed and its application to the proposed information-theoretical approaches is the scope of the future research.

Finally, the proposed procedure is response-adaptive rather than response-adaptive randomised. However, the designs studied in this work serve as a cornerstone to investigate the properties of the proposed measures and to construct the randomised procedure based on them. The response-adaptive randomised information-theoretic procedures that allow to tackle the PCA-power balance are to be explored further.

## Appendix A Supplementary data

MMC S1 Supplementrary material includes the proof of the theorem, the details of the calibration procedure, and extended set of operating characteristics of the proposed designs.

MMC S2

## References

[b1] Azriel D., Mandel M., Rinott Y. (2011). The treatment versus experimentation dilemma in dose finding studies. J. Statist. Plann. Inference.

[b2] Berry D.A. (1978). Modified two-armed bandit strategies for certain clinical trials. J. Amer. Statist. Assoc..

[b3] Cover T.M., Thomas J.A. (2012). Elements of Information Theory.

[b4] Djulbegovic B. (2009). The paradox of equipoise: the principle that drives and limits therapeutic discoveries in clinical research. Cancer Control.

[b5] Hu F., Rosenberger W.F. (2006). The Theory of Response-adaptive Randomization in Clinical Trials.

[b6] Kelbert M., Mozgunov P. (2015). Asymptotic behaviour of the weighted renyi, tsallis and Fisher entropies in a Bayesian problem. Eur. Math. J..

[b7] Kelbert M., Mozgunov P. (2015). Shannon’s differential entropy asymptotic analysis in a Bayesian problem. Math. Commun..

[b8] Lin R., Coleman R.L., Yuan Y. (2020). Top: time-to-event Bayesian optimal phase II trial design for cancer immunotherapy. JNCI: J. Nat. Cancer Inst..

[b9] Lin R., Yuan Y. (2020). Time-to-event model-assisted designs for dose-finding trials with delayed toxicity. Biostatistics.

[b10] MacKay D.J. (2003). Information Theory, Inference, and Learning Algorithms.

[b11] McCullagh P., Nelder J. (1989). Generalized Linear Model.

[b12] Mozgunov P., Jaki T. (2019). An information-theoretic phase I-II design for molecularly targeted agents that does not require an assumption of monotonicity. J. R. Stat. Soc. Ser. C. Appl. Stat..

[b13] Mozgunov P., Jaki T. (2020). An information-theoretic approach for selecting arms in clinical trials. J. R. Stat. Soc. Ser. B.

[b14] Rosenberger W.F. (1999). Randomized play-the-winner clinical trials: review and recommendations. Control. Clin. Trials.

[b15] Sebastiani P., Wynn H.P. (2000). Maximum entropy sampling and optimal Bayesian experimental design. J. R. Stat. Soc. Ser. B Stat. Methodol..

[b16] Sebastiani P., Wynn H.P. (2001). Experimental design to maximize information. AIP Conf. Proc..

[b17] Suhov Y., Stuhl I., Sekeh S.Y., Kelbert M. (2016). Basic inequalities for weighted entropies. Aequationes Math..

[b18] Upton G.J. (1992). Fisher’s exact test. J. R. Stat. Soc. Ser. A.

[b19] Villar S.S., Bowden J., Wason J. (2015). Multi-armed bandit models for the optimal design of clinical trials: benefits and challenges. Stat. Sci. Rev. J. Inst. Math. Stat..

[b20] Villar S.S., Bowden J., Wason J. (2015). Response-adaptive randomization for multi-arm clinical trials using the forward looking gittins index rule. Biometrics.

[b21] Villar S.S., Rosenberger W.F. (2018). Covariate-adjusted response-adaptive randomization for multi-arm clinical trials using a modified forward looking gittins index rule. Biometrics.

[b22] Williamson S.F., Jacko P., Villar S.S., Jaki T. (2017). A Bayesian adaptive design for clinical trials in rare diseases. Comput. Statist. Data Anal..

