# Supplementary material for: Response adaptive designs for Phase II trials with binary endpoint based on context-dependent information measures
Source: Comput Stat Data Anal. 2021 Jun;158:107187. doi: 10.1016/j.csda.2021.107187 (PMC7985674; doi:10.1016/j.csda.2021.107187)
Supplement: MMC S1 — Supplementrary material includes the proof of the theorem, the details of the calibration procedure, and extended set of operating characteristics of the proposed designs. [file mmc1.pdf]

Supplementary Materials

1. Proof of the theorem

The normalizing constants in the weight function (8) has the form

$$\bar{\Lambda}(\gamma, x, n, \upsilon, \beta, \kappa) = \frac{\mathbb{B}(x+\upsilon+1, n-x+\beta-\upsilon+1)}{\mathbb{B}(x+\upsilon+\gamma n^{\kappa}+1, n-x+\beta-\upsilon+n^{\kappa}-\gamma n^{\kappa}+1)} \tag{22}$$

where $\mathbb{B}(x, y)$ is the Beta function. We denote by $\psi^{(0)}(x) = \psi(x)$ the digamma function

$$\psi(x) = \frac{\mathrm{d}}{\mathrm{d}x}\log\Gamma(x) \tag{23}$$

where $\Gamma(x)$ is a Gamma function.

Recall the asymptotics of the digamma functions

$$\psi(x+1) = \log(x) + \frac{1}{2x} + O\left(\frac{1}{x^2}\right) \text{ as } x \to \infty \tag{24}$$

and Stirling's formula

$$n! = \sqrt{2\pi n}\left(\frac{n}{e}\right)^n \left(1 + \frac{1}{12n} + O\left(\frac{1}{n^2}\right)\right) \text{ as } n \to \infty. \tag{25}$$

1.1. Theorem 1

Expressions for the standard Renyi, Tsallis and Fisher DE of r.v. $Z_x^{(n)}$ with PDF (7) can be found in [4].

- Renyi entropy

  Consider the integral:

$$\int_0^1 \phi_\kappa^{(n)} \left(f_x^{(n)}\right)^\nu dp \overset{n\to\infty}{\longrightarrow} U_1 U_2 U_3$$

  where

$$U_1 = \frac{\Gamma(\nu\alpha n + \nu\upsilon + \gamma n^{\kappa} + 1)\Gamma(\nu(1-\alpha)n + \nu(\beta-\upsilon) + (1-\gamma)n^{\kappa} + 1)}{\Gamma(\nu n + n^{\kappa} + \nu\beta + 2)},$$

$$U_2 = \left(\frac{\Gamma(n+\beta+2)}{\Gamma(\alpha n+\upsilon+1)\Gamma((1-\alpha)n+\beta-\upsilon+1)}\right)^{\nu-1},$$

$$U_3 = \frac{\Gamma(n + n^\kappa + \beta + 2)}{\Gamma(\alpha n + \gamma n^\kappa + \upsilon + 1)\Gamma((1-\alpha)n + (1-\gamma)n^\kappa + \beta - \upsilon + 1)}.$$

Above the fact that $\lim_{n\to\infty} \frac{x}{n} = \alpha$ is used and replaced x with αn in U_1, U_2, U_3. Note that $H_\nu^{\phi_\kappa}(f_x^{(n)}) = \frac{1}{1-\nu}\log U_1 U_2 U_3$. Applying Stirling's formula and Taylor series expansion for logarithms we get:

$$\begin{aligned}
\log U_1 \simeq \frac{1}{2}\log\left(\frac{2\pi\alpha(1-\alpha)}{\nu n}\right) + \\
+ (\nu\alpha n + \gamma n^\kappa)\left(\log(\alpha) + \sum_{i=1}^{\infty}\frac{(-1)^{i-1}}{i}\left(\frac{\gamma}{\nu\alpha}\right)^i n^{i\kappa - i}\right) + \\
+(\nu(1-\alpha)n+(1-\gamma)n^\kappa)\left(\log(1-\alpha) + \sum_{i=1}^{\infty}\frac{(-1)^{i-1}}{i}\left(\frac{1-\gamma}{\nu(1-\alpha)}\right)^i n^{i\kappa-i}\right) - \\
-(\nu n + n^\kappa)\sum_{i=1}^{\infty}\frac{(-1)^{i-1}}{i}\left(\frac{1}{\nu}\right)^i n^{i\kappa-i} + \nu\upsilon\log(\alpha) + \nu(\beta-\upsilon)\log(1-\alpha);
\end{aligned}$$

$$\begin{aligned}
\log U_2 \simeq (\nu-1)\bigg(\frac{1}{2}\log\left(\frac{n}{2\pi\alpha(1-\alpha)}\right) - \alpha n\log(\alpha) - \\
-(1-\alpha)n\log(1-\alpha) - \upsilon\log(\alpha) - (\beta-\upsilon)\log(1-\alpha)\bigg);
\end{aligned}$$

$$\begin{aligned}
\log U_3 \simeq -\bigg(\frac{1}{2}\log\left(\frac{2\pi\alpha(1-\alpha)}{n}\right) + \\
+ (\alpha n + \gamma n^\kappa)\left(\log(\alpha) + \sum_{i=1}^{\infty}\frac{(-1)^{i-1}}{i}\left(\frac{\gamma}{\alpha}\right)^i n^{i\kappa-i}\right) + \\
+((1-\alpha)n + (1-\gamma)n^\kappa)\left(\log(1-\alpha) + \sum_{i=1}^{\infty}\frac{(-1)^{i-1}}{i}\left(\frac{1-\gamma}{1-\alpha}\right)^i n^{i\kappa-i}\right) - \\
-(n+n^\kappa)\sum_{i=1}^{\infty}\frac{(-1)^{i-1}}{i}n^{i\kappa-i} + \upsilon\log(\alpha) + (\beta-\upsilon)\log(1-\alpha)\bigg).
\end{aligned}$$

Combining all terms together and removing all terms that decay gives the formula (10).

Proposition 1. *For any fixed large n,*

$$\lim_{\nu \to 1}(H_\nu^{\phi_\kappa}(f_x^{(n)})) = h^{\phi_\kappa}(f_x^{(n)})). \tag{26}$$

Proof of the Proposition 1. From the definition of the Renyi WDE (5) it follows that we need to show that $\frac{\partial}{\partial \nu}((1-\nu)H_\nu^{\phi_\kappa}(f_x^{(n)}))|_{(\nu=1)} = -h^{\phi_\kappa}(f_x^{(n)})$, for any fixed large n. From the Theorem (1) for large n:

$$(1-\nu)H_\nu^{\phi_\kappa}(f_x^{(n)}) = \frac{1}{2}(1-\nu)\log\left(\frac{2\pi\alpha(1-\alpha)}{n}\right)+\omega(\nu,\alpha,\kappa,n,\gamma)-\frac{1}{2}\log(\nu).$$

Differentiation yields:

$$\frac{\partial}{\partial \nu}\left((1-\nu)H_\nu^{\phi_\kappa}(f_x^{(n)})\right) = -\frac{1}{2}\log\left(\frac{2\pi e\alpha(1-\alpha)}{n}\right) + \frac{\partial}{\partial \nu}\omega(\nu,\alpha,\kappa,n,\gamma),$$

$$\begin{aligned}\frac{\partial}{\partial \nu}\omega(\nu,\alpha,\kappa,n,\gamma) = &\sum_{i=1}^{\lfloor\frac{1}{1-\kappa}\rfloor}\frac{(-1)^{i-1}}{i}\Bigg(-\frac{\gamma^i}{\alpha^{i-1}}\frac{(i-1)}{\nu^i}- \\ &-\frac{(1-\gamma)^i}{(1-\alpha)^{i-1}}\frac{(i-1)}{\nu^i}+\frac{(i-1)}{\nu^i}\Bigg)n^{i\kappa-i+1}+ \\ &+\sum_{i=1}^{\lfloor\frac{\kappa}{1-\kappa}\rfloor}\frac{(-1)^{i-1}}{i}\Bigg(-\frac{\gamma^{i+1}}{\alpha^i}\frac{i}{\nu^{i+1}}-\frac{(1-\gamma)^{i+1}}{(1-\alpha)^i}\frac{i}{\nu^{i+1}}+\frac{i}{\nu^{i+1}}\Bigg)n^{(i+1)\kappa-i}.\end{aligned}$$

Inserting $\nu = 1$ in the expression above yields:

$$\begin{aligned}\frac{\partial}{\partial \nu}\omega(\nu,\alpha,\kappa,n,\gamma) = &\sum_{i=1}^{\lfloor\frac{1}{1-\kappa}\rfloor}\frac{(-1)^{i-1}}{i}\Bigg(-\frac{\gamma^i}{\alpha^{i-1}}(i-1)- \\ &-\frac{(1-\gamma)^i}{(1-\alpha)^{i-1}}(i-1)+(i-1)\Bigg)n^{i\kappa-i+1}+ \\ &+\sum_{i=1}^{\lfloor\frac{\kappa}{1-\kappa}\rfloor}\frac{(-1)^{i-1}}{i}\Bigg(-\frac{\gamma^{i+1}}{\alpha^i}i-\frac{(1-\gamma)^{i+1}}{(1-\alpha)^i}i+i\Bigg)n^{(i+1)\kappa-i}.\end{aligned} \tag{27}$$

For $0 < \kappa < \frac{1}{2}$, $\frac{\partial}{\partial \nu}\omega(\nu,\alpha,\kappa,n,\gamma) = 0$ and the equation (26) holds. For $\frac{1}{2} \leq \kappa < 1$, note, that the first term in the first series of (27) is equal to

zero and $\forall\, i,\ (i+1)\kappa - i > 0 \Leftrightarrow j\kappa - j + 1 > 0$ where $j = i + 1$. Thus, $\forall \kappa \in (\frac{1}{2}, 1),\ \lfloor \frac{1}{1-\kappa} \rfloor - \lfloor \frac{\kappa}{1-\kappa} \rfloor = 1$ and we can combine two series of (27) together and get:

$$\frac{\partial}{\partial \nu}\omega(\nu,\alpha,\kappa,n,\gamma) = \sum_{i=2}^{\lfloor \frac{1}{1-\kappa} \rfloor} \left(\frac{(-1)^{i-1}}{i} + \frac{(-1)^{i-2}}{i-1} \right)$$

$$\left(-\frac{\gamma^i}{\alpha^{i-1}}(i-1) - \frac{(1-\gamma)^i}{(1-\alpha)^{i-1}}(i-1) + (i-1) \right) n^{i\kappa - i + 1} =$$

$$\sum_{i=2}^{\lfloor \frac{1}{1-\kappa} \rfloor} \frac{(-1)^{i-1}}{i} \left(\frac{\gamma^i}{\alpha^{i-1}} + \frac{(1-\gamma)^i}{(1-\alpha)^{i-1}} - 1 \right) n^{i\kappa - i + 1}.$$

Combining all terms together the formula (26) immediately follows for $\kappa \in (0, 1)$.

- Tsallis entropy

  The proof is straightforward since $T_q^{\phi_\kappa}(f_x^{(n)}) = \frac{1}{q-1}\left(1 - \exp\left((1-q)H_q^{\phi_\kappa}(f_x^{(n)})\right)\right)$. Using asymptotics for $H_q^{\phi_\kappa}(f_x^{(n)})$ from the Theorem 1 gives the formula (11).

  Proposition 2. *For any fixed large n,*

$$\lim_{q \to 1}(T_q^{\phi_\kappa}(f_x^{(n)})) = h^{\phi_\kappa}(f_x^{(n)})). \tag{28}$$

  Proof of the Proposition 2. From the definition of the Tsallis WDE (6) it follows that we need to show that $\frac{\partial}{\partial \nu}((q-1)T_q^{\phi_\kappa}(f_x^{(n)}))|_{(q=1)} = h^{\phi_\kappa}(f_x^{(n)})$, for any fixed large n. From the Theorem (1), for large n,

$$(q-1)T_q^{\phi_\kappa}(f_x^{(n)}) = \left(1 - \frac{1}{\sqrt{q}}\left(\frac{2\pi\alpha(1-\alpha)}{n}\right)^{\frac{1-q}{2}} exp\Big\{\omega(q,\alpha,\kappa,n,\gamma)\Big\}\right),$$

$$\frac{\partial}{\partial q}\left((q-1)T_q^{\phi_\kappa}(f_x^{(n)})\right) = -exp\Big\{\omega(q,\alpha,\kappa,n,\gamma)\Big\}\Bigg[\left(\frac{\partial}{\partial q}\omega(q,\alpha,\kappa,n,\gamma)\right)$$

$$\frac{1}{\sqrt{q}}\left(\frac{2\pi\alpha(1-\alpha)}{n}\right)^{\frac{1-q}{2}} + \frac{\partial}{\partial q}\left(\frac{1}{\sqrt{q}}\left(\frac{2\pi\alpha(1-\alpha)}{n}\right)^{\frac{1-q}{2}}\right)\Bigg].$$

  Using formula (27) and inserting $q = 1$ in the expression above gives the formula (28).

- Fisher entropy

  Consider the Fisher Information

$$I^{\phi_\kappa}(\alpha) = \int_0^1 \phi_\kappa^{(n)} \left(\frac{\partial}{\partial\alpha}\log f_x^{(n)}\right)^2 f_x^{(n)} \mathrm{d}p, \tag{29}$$

  where

$$\left(\frac{\partial}{\partial\alpha}\log f_x^{(n)}\right)^2 \simeq n^2\Big(\log^2(p) + \log^2(1-p) - 2\log(p)\log(1-p) + \\ + (\psi((1-\alpha)n+\beta-\upsilon+1) - \psi(\alpha n+\upsilon+1))^2 + \\ + 2(\log(p) - \log(1-p))(\psi((1-\alpha)n+\beta-\upsilon+1) - \psi(\alpha n+\upsilon+1)\Big).$$

  In the above expression the fact that $\lim_{n\to\infty}\frac{x}{n} = \alpha$ is used. Integral (29) can be found explicitly (see [4]). After simplification,

$$I^{\phi_\kappa}(\alpha) = n^2\Big[\psi'(\alpha n+\gamma n^\kappa+\upsilon+1)+\psi'((1-\alpha)n+(1-\gamma)n^\kappa+\beta-\upsilon+1)\Big]+ \\ + n^2\Big[(\psi(\alpha n+\gamma n^\kappa+\upsilon+1) - \psi((1-\alpha)n+(1-\gamma)n^\kappa+\beta-\upsilon+1)) - \\ - (\psi(\alpha n+\upsilon+1) - \psi((1-\alpha)n+\beta-\upsilon+1))\Big]^2$$

  where $\psi'(x)$ is a derivative of the Digamma function. Using asimptotics for the Digamma function, given that β and υ are constants,

$$I^{\phi_\kappa}(\alpha) \simeq n\left(\frac{1}{\alpha+\gamma n^{\kappa-1}} + \frac{1}{(1-\alpha)+(1-\gamma)n^{\kappa-1}}\right) - \\ - \frac{1}{2}\left(\frac{1}{(\alpha+\gamma n^{\kappa-1})^2} + \frac{1}{((1-\alpha)+(1-\gamma)n^{\kappa-1})^2}\right) + \\ + \left[n\left(\log\left(1+\frac{\gamma}{\alpha}n^{\kappa-1}\right) - \log\left(1+\frac{1-\gamma}{1-\alpha}n^{\kappa-1}\right)\right) + \\ + \frac{(1-2\alpha)+(1-2\gamma)n^{\kappa-1}}{2(\alpha+\gamma n^{\kappa-1})((1-\alpha)+(1-\gamma)n^{\kappa-1})} + \frac{2\alpha-1}{2\alpha(1-\alpha)}\right]^2. \tag{30}$$

Applying Taylor series expansion for logarithms,

$$
\begin{aligned}
I^{\phi_\kappa}(\alpha) \simeq n\left(\frac{1}{\alpha+\gamma n^{\kappa-1}}+\frac{1}{(1-\alpha)+(1-\gamma)n^{\kappa-1}}\right)- \\
-\frac{1}{2}\left(\frac{1}{(\alpha+\gamma n^{\kappa-1})^2}+\frac{1}{((1-\alpha)+(1-\gamma)n^{\kappa-1})^2}\right)+ \\
+\Bigg[\sum_{m=1}^{\infty}\frac{(-1)^{m-1}}{m}\left(\left(\frac{\gamma}{\alpha}\right)^m-\left(\frac{1-\gamma}{1-\alpha}\right)^m\right)n^{m\kappa-m+1}+ \\
\frac{(1-2\alpha)+(1-2\gamma)n^{\kappa-1}}{2(\alpha+\gamma n^{\kappa-1})((1-\alpha)+(1-\gamma)n^{\kappa-1})}+\frac{2\alpha-1}{2\alpha(1-\alpha)}\Bigg]^2,
\end{aligned}
\tag{31}
$$

$$
\begin{aligned}
I^{\phi_\kappa}(\alpha) \simeq n\left(\frac{1}{\alpha(1-\alpha)}\right)-\frac{1}{2}\left(\frac{1}{\alpha^2}+\frac{1}{(1-\alpha)^2}\right)+ \\
+\left(\sum_{m=1}^{\infty}\frac{(-1)^{m-1}}{m}\left(\left(\frac{\gamma}{\alpha}\right)^m-\left(\frac{1-\gamma}{1-\alpha}\right)^m\right)n^{m\kappa-m+1}\right)^2+ \\
+\left(\frac{(1-2\alpha)+(1-2\gamma)n^{\kappa-1}}{2(\alpha+\gamma n^{\kappa-1})((1-\alpha)+(1-\gamma)n^{\kappa-1})}+\frac{2\alpha-1}{2\alpha(1-\alpha)}\right)^2+ \\
+2\sum_{m=1}^{\infty}\frac{(-1)^{m-1}}{m}\left(\left(\frac{\gamma}{\alpha}\right)^m-\left(\frac{1-\gamma}{1-\alpha}\right)^m\right) \\
n^{m\kappa-m+1}\Bigg(n^{\kappa-1}\frac{1-2\gamma}{2(\alpha+\gamma n^{\kappa-1})((1-\alpha)+(1-\gamma)n^{\kappa-1})}+ \\
+\frac{1-2\alpha}{2(\alpha+\gamma n^{\kappa-1})((1-\alpha)+(1-\gamma)n^{\kappa-1})}+\frac{2\alpha-1}{2\alpha(1-\alpha)}\Bigg).
\end{aligned}
\tag{32}
$$

Note, that the fourth term in (32) tends to zero and the last two terms in the last parenthesis cancel each out as n tends to infinity. Expanding the first squared expression,

$$
\begin{aligned}
\left(\sum_{m=1}^{\infty}\frac{(-1)^{m-1}}{m}\left(\left(\frac{\gamma}{\alpha}\right)^m-\left(\frac{1-\gamma}{1-\alpha}\right)^m\right)n^{m\kappa-m+1}\right)^2 \simeq \\
\simeq \sum_{m_1,m_2\in\mathbb{N}:\, m_1+m_2\le\lfloor\frac{2}{1-\kappa}\rfloor}\frac{(-1)^{m_1+m_2-2}}{m_1m_2}\left(\left(\frac{\gamma}{\alpha}\right)^{m_1}-\left(\frac{1-\gamma}{1-\alpha}\right)^{m_1}\right) \\
\left(\left(\frac{\gamma}{\alpha}\right)^{m_2}-\left(\frac{1-\gamma}{1-\alpha}\right)^{m_2}\right)n^{(m_1+m_2)\kappa-(m_1+m_2)+2}.
\end{aligned}
$$

After multiplication the last term in (32) tends to:

$$2\sum_{m=1}^{\infty}\frac{(-1)^{m-1}}{m}\left(\left(\frac{\gamma}{\alpha}\right)^m-\left(\frac{1-\gamma}{1-\alpha}\right)^m\right)$$
$$n^{m\kappa-m+1}\left(n^{\kappa-1}\frac{1-2\gamma}{2(\alpha+\gamma n^{\kappa-1})((1-\alpha)+(1-\gamma)n^{\kappa-1})}\right)\simeq$$
$$\simeq 2\sum_{m=1}^{\lfloor\frac{\kappa}{1-\kappa}\rfloor}\left(\left(\frac{\gamma}{\alpha}\right)^m-\left(\frac{1-\gamma}{1-\alpha}\right)^m\right)\left(\frac{1-2\gamma}{2\alpha(1-\alpha)}\right)n^{(m+1)\kappa-m}.$$

Combining all terms together we obtain the formula (12).

2. Calibration

Below, we provide the details on calibration procedure described in Section 3.2, namely, calibration of prior parameter E, cut-off parameter δ and additional parameters q and ν.

2.1. *Calibration of prior distribution*

For each new design, the growth of E leads to an improvement of power and deterioration of PCA. The major problem when calibrating the E was type I error, as for various values of κ the effect of E on type I error is unpredictable (see Figure 5). To solve the problem a procedure was adjusted manually via a cut-off parameter δ. In general, for most of the designs, the power-PCA balance is more likely to be shifted towards power. Thus, initially, we were interested in values of $E \leq 10$ in order to improve PCA in exchange for a minor price in power.

Consider the AF, the numerical results for calibration of which are given in Table 2. At first, the experiment is conducted with different sets of parameters

Table 2: The total number of values of the parameter $\kappa \in \{0.1, 0.11, ..., 0.9\}$ for which type I error rate is higher than 10% calculated for the AF with fixed E, δ.

δ \ E	1	2	3	4	5	**6**	7	8	9	10
10%	68	69	63	52	38	30	25	14	15	10
9.5%	45	44	36	25	15	11	5	1	0	0
9%	43	38	28	15	8	4	0	0	0	0
8.5%	33	27	11	6	1	**0**	0	0	0	0

$\kappa \in \{0.1, 0.11, \ldots, 0.9\}, E \in \{1, 2, \ldots, 10\}, \delta \in \{0.085, \ldots, 0.1\}$. Then the number of designs for which type I error is violated is calculated. The goal is to choose the smallest value of E such that for all values of κ type I error rate is smaller than 10%.

$(a) \quad \delta = 0.1$

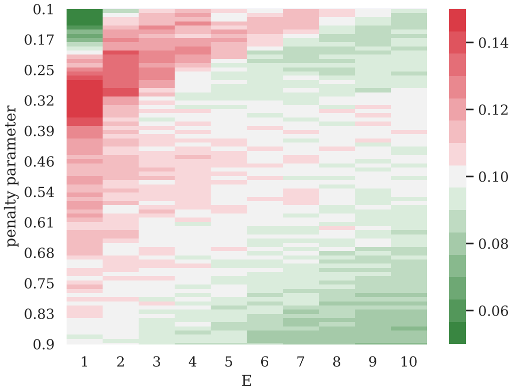

$(b) \quad \delta = 0.085$

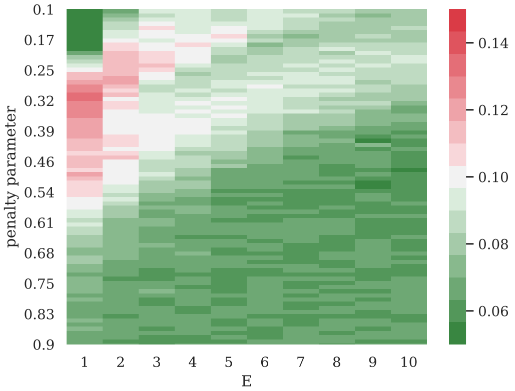

Figure 5: The effect of E on type I error rate for the AF for the designs with $\delta = 0.085$ and $\delta = 0.1$. Type I error, calculated for all of the designs with various penalty parameter $\kappa \in \{0.1, 0.11, \ldots, 0.99\}$ (top to bottom) and prior parameter $E \in \{1, 2, \ldots, 10\}$ (left to right), is represented by a color in a range from 15% (brightest red) to 5% (darkest green), with target values less than 10% (white).

For instance, for the AF several pairs of parameters E and cut-off δ can be chosen: $E = 9$ with $\delta = 9.5\%$, $E = 7$ with $\delta = 9\%$, and $E = 6$ with $\delta = 8.5\%$. Since we are interested in smaller values of E, the pair $E = 6$, $\delta = 0.085$ is chosen. Values of E and δ for other entropy criteria were calibrated following the similar logic.

2.2. Calibration of parameters for Fisher and Tsallis entropies

Further, we present details on the calibration of q for the exact criterion based on Tsallis entropy and ν for the exact criterion based on Renyi entropy. The calibration of these parameters was conducted comparing the designs with $\kappa \in \{0.1, 0.2, \ldots, 0.9\}$ with already calibrated values of $E = 9$ and $E = 8$, for Tsallis and Renyi respectively.

During a pairwise comparison of the designs with different values of additional parameters q and ν for the Tsallis and Renyi designs, respectively, in terms of power and PCA, it was found that the designs with $q = 0.35$ and $\nu = 0.75$ work slightly better or comparably good in terms of Power and PCA among the designs with different values of q or ν. The operating characteristics for $q \in \{0.1, 0.35, 0.9\}$ and $\nu \in \{0.1, 0.75, 0.9\}$, for Tsallis and Renyi respectively and $\kappa \in \{0.1, 0.5, 0.9\}$ are given in Figure 6. Figure 6 also illustrates that q and ν have smaller effect on the operating characteristics in comparison to κ. For this reason, more rigorous calibration might be excessive.

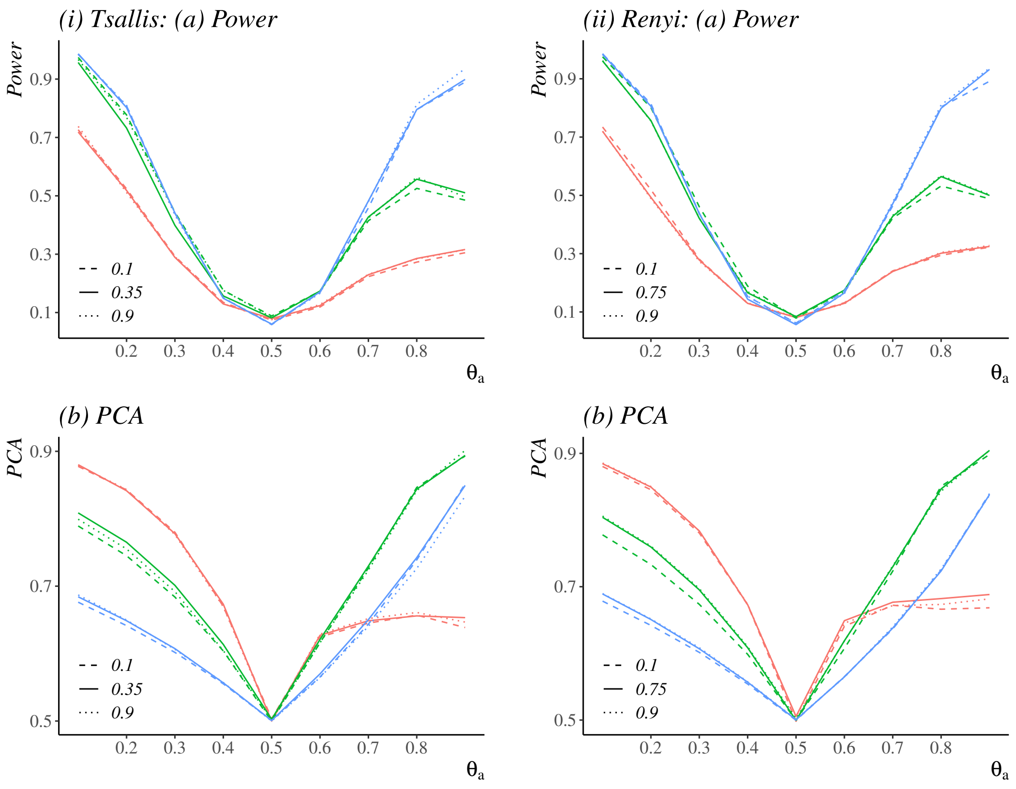

Figure 6: The effect of q and ν, for Tsallis and Renyi respectively, on operating characteristics: solid line – $q = 0.35$, $\nu = 0.75$; dashed line – $q = \nu = 0.1$; dotted line – $q = \nu = 0.9$; ; red line, $\kappa = 0.1$; green line, $\kappa = 0.5$; blue line, $\kappa = 0.9$.

3. Operating characteristics

Below, the numerical results for the designs based on different information measures are given. First, the operating characteristics of interest are compared for the Renyi and Tsallis entropy criteria. Afterwards, we address the problem with the "asymmetrical" designs. Finally, an alternative operating characteristic, namely, probability of correct selections (PCS), for comparison of the designs is considered.

The illustration of how different values of $\kappa \in \{0.1, 0.5, 0.9\}$ influence operating characteristics for Renyi criterion in comparison to Tsallis criterion are presented in Figure 7. The results for Renyi and Tsallis criteria based designs are comparable in terms of both power and PCA for different scenarios (Figure 7) and on average in comparison to FR (Figure 8).

Note, that both designs are asymmetrical in terms of both power and PCA for small values of $\kappa \in \{0.1, 0.2\}$. Designs asymmetrical in terms of PCA are not considered in the analysis for a reason described below.

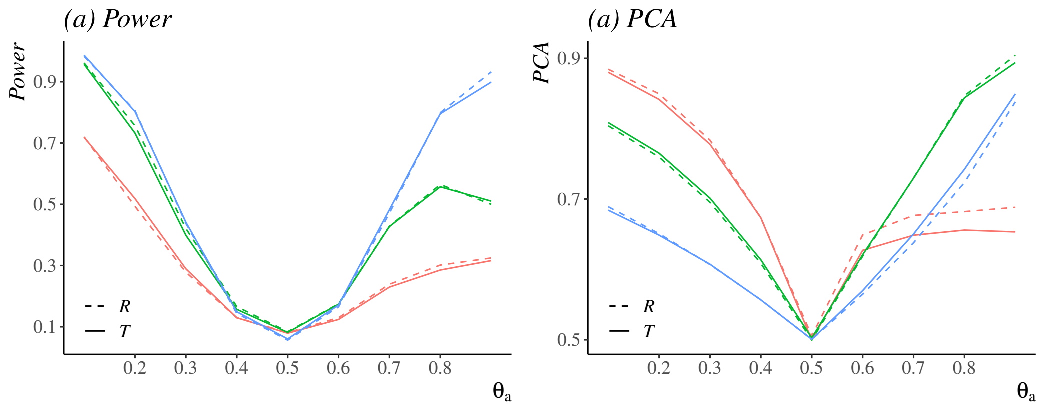

Figure 7: Comparison of Renyi criterion with respect to Tsallis criterion in terms of power and PCA: solid line – Tsallis criteria; dashed line – Renyi criteria; red line, $\kappa = 0.1$; green line, $\kappa = 0.5$; blue line, $\kappa = 0.9$.

For the Tsallis criterion in terms of PCA for $\theta_a < 0.5$ $T0.1$ outperforms $T0.5$, with average difference of 9.7%, and $T0.5$ outperforms $T0.9$, with average difference of 7.1%. In contrast to the common positive effect of κ on PCA, $T0.1$ is outperformed by $T0.5$ under scenarios with $\theta_a > 0.6$ by an average of 12.6%.

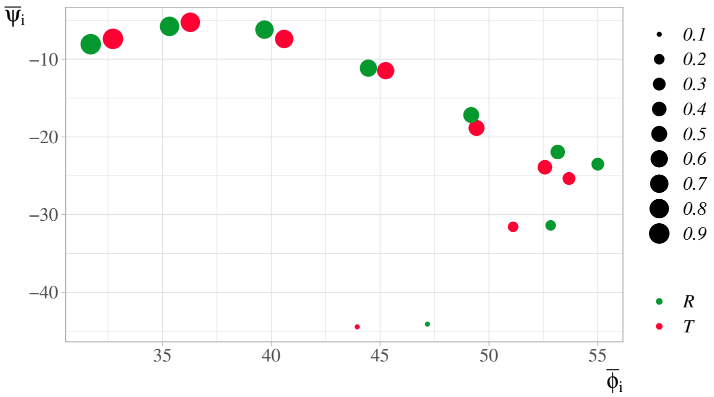

Figure 8: Mean percentage power loss $\bar{\psi}_i$ vs mean percentage PCA gain $\{\phi\}_i$ for design i for Tsallis (red dots) and Renyi (green dots) criteria in comparison to FR approach. The size of dots corresponds to the value of κ: smallest dot, $\kappa = 0.1$; largest dot, $\kappa = 0.9$.

3.1. Designs asymmetric in terms of power

In the Section 3.3. it was stated that the drawback in terms of power for the $AS0.5$ design for the scenarios with $\theta_a > 0.5$ was caused by the form of the chosen weight function reflecting the interest in the outcomes close to $\gamma = 0.999$. To describe this drawback the probability of allocating x of 75 patients to less efficacious arm B was calculated as a share of iterations for which this event took place (Figure 9). Recall, that the total number of iterations is $10,000$, $\theta_a = 0.9$, $\theta_b = 0.5$.

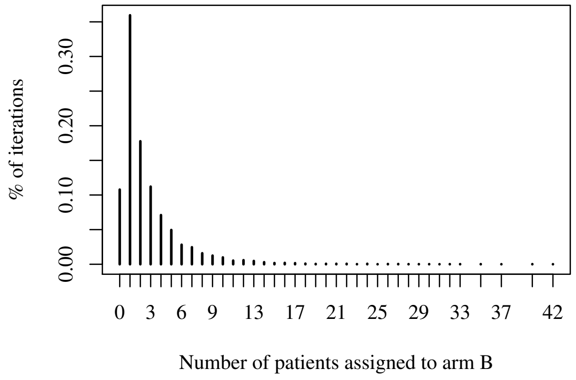

Figure 9: Number of patients assigned to a less efficacious arm B and corresponding frequencies for $AS0.5$ in the scenario $\theta_a = 0.9$.

If the penalty parameter κ is small, then with a high probability by the end of the experiment, less than 10 out of 75 patients will receive the inferior treatment. In particular, for the $AS0.5$ design with the scenario $\theta_a = 0.9$, with the probability of 83.7% 5 or less patients were assigned to treatment arm B, with the probability of 11.4% no patients being assigned to arm B. Therefore, in a high proportion of simulated trials, there might not be enough information to obtain statistically significant results. Although Fisher's exact test is known to be valid even for small sample sizes, it is implemented at the end of the procedure and does not account for all the information collected during the sequential trials, i.e. the fact that during the experiment only several patients received the treatment from one arm tells us that the design considers another arm as a more efficacious. Presumably, this issue

would be resolved if the proposed response-adaptive design will be randomised, which is the scope of further research. The significance level for Fisher's test at the end of the experiment can be made more "flexible", so that for small values of κ in a way that it would account for the changes in information gains. However, a more accurate study is required. Note, that $S0.5$ and $T0.5$ were found to be highly asymmetrical in terms of power for the same reason.

3.2. Designs asymmetric in terms of PCA

To describe the drawback in terms of PCA for the $T0.1$ design for the scenarios with $\theta_a > 0.5$, the probability of allocating x of 75 patients to a superior arm A was calculated as a share of iterations for which this event happened. The probabilities were calculated for the scenario with $\theta_a = 0.9$, $\theta_b = 0.5$. The results are given in Figure 10.

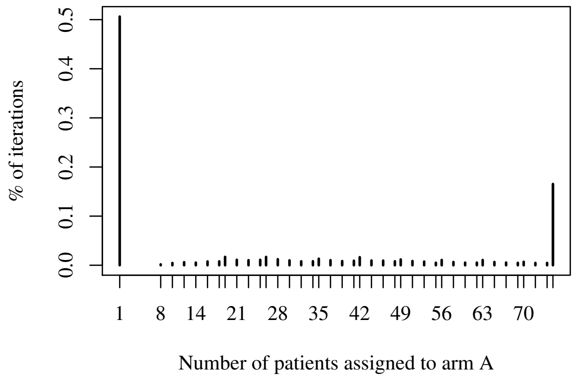

Figure 10: Number of patients assigned to a truly superior arm A and corresponding frequencies for $T0.1$ in the scenario $\theta_a = 0.9$. Note, in the scenarios $\theta_a \in 0.6, 0.7, 0.8$ analogous plots look similarly.

It follows that for $T0.1$ the effect of κ is so extreme that the changes in information gain are made slowly, as negative responses are not penalized enough. So, when the first patient gets assigned to arm B with probability 0.5, since the priors for both treatment arms are equal, and responds positively with probability $\theta_b = 0.5$, the design sticks to it, resulting in a situation when for each of $\theta_a > 0.5$ with probability of 17% no patients were assigned to a superior arm A.

This drawback can be adjusted manually, e.g. more than one patient should be randomly allocated to the treatment arm. However until a more detailed study is conducted the designs with small values of κ, which are highly asymmetrical in terms of PCA, will not be considered in a set of competing designs.

3.3. Dynamic programming approaches

To support the choice of $p = 0.9$ for the CRDP design, we provide the averaged operating characteristics of several specifications of CRDP designs with $p \in \{0.6, 0.7, 0.8, 0.9\}$ and $l/n \in \{0.1, 0.15, 0.25, 0.35\}$ in Figure 11. Considering the designs with a balance shifted towards power, no significant difference was found between the designs with $l = 0.35n$ and various values of p, for the designs with $l = 0.35n$ the comparison is followed by a scenario-by-scenario comparison. Considering the designs with a balance shifted towards PCA, in terms of averaged characteristics, the advantage in terms of PCA when choosing $p = 0.9$ is more prominent in comparison to designs shifted towards power.

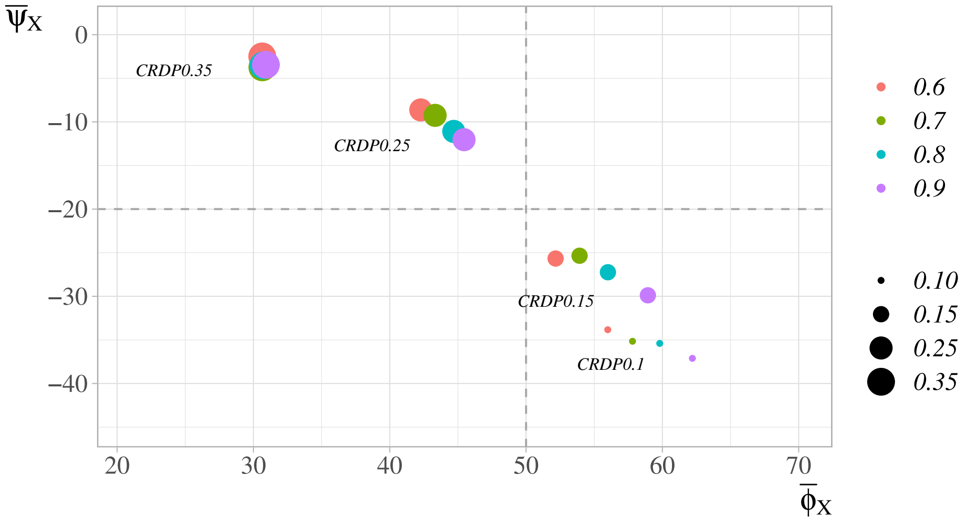

Figure 11: An average percentage power loss $\bar{\psi}_i$ vs an average percentage PCA gain $\bar{\phi}_i$ for CRDP designs with $p \in \{0.6, 0.7, 0.8, 0.9\}$ and $l/n \in \{0.1, 0.15, 0.25, 0.35\}$. The size of dots corresponds to the value of l/n: smallest dot, $l/n = 0.1$; largest dot, $l/n = 0.35$. The color of dots corresponds to the value of p: red dot, $p = 0.6$; green dot, $p = 0.7$; blue dot, $p = 0.8$; violet dot, $p = 0.6$. The designs favouring the power are represented by the dots which lie above a horizontal dashed line; the designs favouring the PCA – to the right of a vertical dashed line.

In terms of scenario-by-scenario comparison (Figure 12), for $CRDP0.1$ with $p = 0.6$ and $p = 0.9$ the average difference in terms of power is 0.6%, and for $CRDP0.25$ with $p = 0.6$ and $p = 0.9$ the average difference in terms of power is 1.5%. For $CRDP0.1$ with $p = 0.6$ and $p = 0.9$ the average difference in terms of PCA is 3.4%, and for $CRDP0.25$ with $p = 0.6$ and $p = 0.9$ the average difference in terms of power is 1.7%.

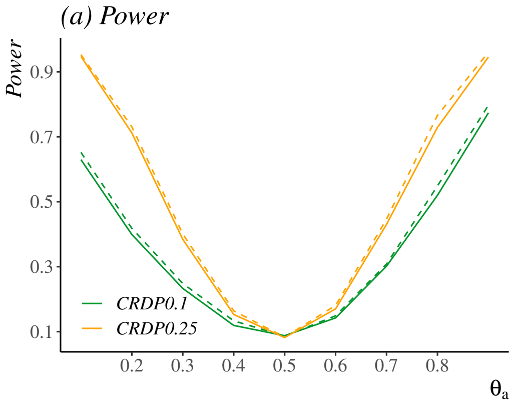

Figure 12: Comparison of $CRDP0.1$ (green line) and $CRDP0.25$ (orange line) with $p = 0.6$ (dashed line) and $p = 0.9$ (solid line) in terms of power and PCA.
